# Gα_i_2 Protein Inhibition Blocks Chemotherapy- and Anti-Androgen-Induced Prostate Cancer Cell Migration

**DOI:** 10.3390/cancers16020296

**Published:** 2024-01-10

**Authors:** Silvia Caggia, Alexis Johnston, Dipak T. Walunj, Aanya R. Moore, Benjamin H. Peer, Ravyn W. Everett, Adegboyega K. Oyelere, Shafiq A. Khan

**Affiliations:** 1 Center for Cancer Research and Therapeutic Development, Clark Atlanta University, 223 James P. Brawley Dr., Atlanta, GA 30314, USA; scaggia@cau.edu (S.C.); moorear1209@gmail.com (A.R.M.); ravyneverett@gmail.com (R.W.E.); 2 School of Chemistry and Biochemistry, Georgia Institute of Technology, 901 Atlantic Drive, Atlanta, GA 30318, USA; ajohnston48@gatech.edu (A.J.); dipak.walunj@chemistry.gatech.edu (D.T.W.); bpeer7@gatech.edu (B.H.P.); 3 Parker H. Petit Institute for Bioengineering and Bioscience, Georgia Institute of Technology, 315 Ferst Dr. NW, Atlanta, GA 30332, USA

**Keywords:** cell migration, chemotherapy, HDACi, Gα_i_2, cancer, metastases

## Abstract

**Simple Summary:**

Chemotherapy is the first line of treatment for malignant tumors. However, recent discoveries have shown that a relapse and the formation of metastatic disease could be facilitated by these treatments. In this study, we show how several chemotherapeutic drugs induced prostate cancer cells migration. Additionally we propose that combination therapy with newly synthesized inhibitors could block the chemotherapy-driven metastatic behavior of prostate cancer cells.

**Abstract:**

We have previously shown that heterotrimeric G-protein subunit alphai2 (Gα_i_2) is essential for cell migration and invasion in prostate, ovarian and breast cancer cells, and novel small molecule inhibitors targeting Gα_i_2 block its effects on migratory and invasive behavior. In this study, we have identified potent, metabolically stable, second generation Gα_i_2 inhibitors which inhibit cell migration in prostate cancer cells. Recent studies have shown that chemotherapy can induce the cancer cells to migrate to distant sites to form metastases. In the present study, we determined the effects of taxanes (docetaxel), anti-androgens (enzalutamide and bicalutamide) and histone deacetylase (HDAC) inhibitors (SAHA and SBI-I-19) on cell migration in prostate cancer cells. All treatments induced cell migration, and simultaneous treatments with new Gα_i_2 inhibitors blocked their effects on cell migration. We concluded that a combination treatment of Gα_i_2 inhibitors and chemotherapy could blunt the capability of cancer cells to migrate and form metastases.

## 1. Introduction

Cancer metastasis, a leading cause of mortality in cancer patients, is a complex process [1]. Metastasis of epithelial cancer cells involves a cascade of biological events resulting in the establishment of macrometastases at distant sites [2,3]. Tumor cell motility is the initial step in the process of invasion and metastasis and is essential for the dissemination of primary tumor cells to local and distant sites [4]. Cancer cell migration is a finely regulated and complex process, during which cells, upon sensing chemotactic stimuli, activate several signal transduction mechanisms which eventually will induce the polymerization of new actin filaments at the leading edge, causing the formation of sheet-like membrane protrusions called lamellipodia [5].

We have recently shown that Gα_i_2 protein, a subunit of the heterotrimeric G-proteins complex, plays an essential role during cell migration and invasion of prostate, ovarian and breast cancer cells [6]. Our studies have also shown that Gα_i_2 acts at two distinct intracellular levels to exert its effects on cell migration. First, its activation through specific GPCRs is required for the induction of cell migration and invasion in response to several chemokines and growth factors [7]. This effect is upstream of the activation of PI3-kinase/Akt pathway. Second, Gα_i_2 protein is required for the formation of lamellipodia at the leading edge of migrating cells. This novel effect of Gα_i_2 does not require activation by GPCRs, and it is downstream or independent of PI3-kinase and Rac1 activation [7]. Since Gα_i_2 plays an essential role in cancer cell migration and invasion, we have synthesized a cohort of small molecule inhibitors which blocks the activation of the Gα_i_2 protein, leading to the inhibition of cell migration and invasion in several cancer types [8].

Prostate cancer is the second most diagnosed cancer and the second leading cause of cancer deaths among American men. According to the American Cancer Society, it was predicted that 288,300 men will be diagnosed and 34,700 men will die of prostate cancer in the US in 2023 [9]. The majority of prostate cancer patients will die not as a result the primary tumor but as a result of the development of metastatic disease [10]. Current treatments for metastatic disease are hormonal therapy and chemotherapy. Androgen deprivation therapy (ADT), based on inhibition of androgen biosynthesis, and/or action is universally accepted as the first-line treatment of symptomatic metastatic prostate cancer [11]. Anti-androgens, such as bicalutamide, nilutamide, flutamide, enzalutamide, apalutamide and darolutamide, work by blocking androgen receptor (AR) in prostate cancer cells [12]. Enzalutamide has been shown to drastically improve the overall survival in metastatic castration-resistant prostate cancer (mCRPC) patients before and after chemotherapy [13,14]. For CRPC patients, docetaxel has been used as a standard chemotherapy regimen. Additionally, combination of ADT and docetaxel has also been introduced to treat hormone sensitive prostate cancers, a therapeutic regimen that has increased the patients overall survival [15]. A class of chemotherapeutic agents, which have shown benefits in the management of hematological malignancies, are histone deacetylase inhibitors (HDACi). The potential of HDACi as therapeutic agents for solid tumors, including prostate cancer, is also being investigated in several pre-clinical and clinical studies [16,17]. We have previously developed very potent HDAC inhibitors conjugated with AR antagonists, which were shown to be highly cytotoxic for prostate cancer cells in vitro and in vivo [18,19].

Recent studies have shown that chemotherapy itself can increase the capability of cancer cells to escape from death and migrate to distant sites to form metastases [20,21,22,23]. The cellular and molecular mechanisms involved in these divergent effects are not well defined. However, these studies suggest that as a response to cytotoxic effects of chemotherapy, the tumor cells may not only develop drug resistance but the altered tumor microenvironment may also induce migratory and invasive behavior in target cells, which may lead to increased metastases [24,25]. Such effects of chemotherapy on migratory and invasive behavior have not been well investigated in prostate cancers.

The current studies were carried out to optimize the first generation Gα_i_2 inhibitors and determine the possible effects of representative chemotherapeutic drugs (taxanes, anti-androgens and HDACi) on cell migration in prostate cancer cells and whether these effects can be blocked by the simultaneous treatment with these novel Gα_i_2 inhibitors.

## 2. Materials and Methods

### 2.1. Chemicals and Reagents

Reagents and solvents for synthesis were purchased from Sigma-Aldrich (St. Louis, MO, USA), VWR International, (Radnor, PA, USA), Combi-blocks and Ambeed and were used as received. Analtech silica gel plates (60 F254) were used for analytical TLC, and plates were visualized using UV light, anisaldehyde and/or iodine stains. Analtech preparative TLC plates (UV254, 2000 μm) and silica gel (200–400 mesh) were used for purification by prep-TLC and column chromatography, respectively. Compounds were characterized using NMR spectra obtained on a Varian-Gemini 400 MHz and Bruker Ascend™ 500 and 700 MHz magnetic resonance spectrometer. ^1^H NMR spectra were recorded in parts per million (ppm) relative to the residual peaks of CHCl_3_ (7.24 ppm) in CDCl_3_ or DMSO-*d*5 (2.49 ppm) in DMSO-*d*6. ^13^C NMR spectra were recorded relative to the CDCl_3_ triplet peak (77.0 ppm) or the DMSO-*d*6 septet (39.7 ppm) and were recorded with complete heterodecoupling. Multiplicities are described using the abbreviation s, singlet; d, doublet; t, triplet; q, quartet; m, multiplet; and app, apparent. MestReNova (version 11.0) was used to process the “fid” files. Low- and high-resolution mass spectra were obtained at the Georgia Institute of Technology mass spectrometry facility (Atlanta, GA, USA).

### 2.2. Synthesis

Details about the synthesis and characterization of the disclosed compounds are available in the Supporting Information.

### 2.3. Cell Culture and Reagents

LNCaP human AR+ (ATCC^®^ CRL-1740™), DU145 (ATCC^®^ HTB-81™) and PC3 human AR− (ATCC^®^ CRL-1435™) prostate cancer cell lines were obtained from the American Type Culture Collection (ATCC) (Rockville, MD, USA). They were maintained as previously described [7,8], and they were tested periodically for mycoplasma contamination using PlasmoTest™—Mycoplasma Detection Kit (InvivoGen, San Diego, CA, USA). Anti-α-tubulin and bovine serum albumin (BSA) were obtained from Sigma-Aldrich (St. Louis, MO, USA). Rat tail collagen and transwell inserts were obtained from BD Biosciences (San Jose, CA, USA). DAPI (4′,6-Diamidino-2-Phenylindole) was purchased from Invitrogen through Thermo Fisher Scientific (Eugene, OR, USA). Rabbit polyclonal anti-Gα_i_2 antibody (ab 157204) was purchased from Abcam (Cambridge, MA, USA). Epidermal growth factor (EGF) was obtained from Life Technologies (Grand Island, NY, USA). The anti-rabbit and anti-mouse immunoglobulins coupled with horseradish peroxidase (IgG-HRP), were obtained from Promega (Madison, WI, USA). Cell culture reagents were purchased from Corning Life Sciences (Tewksbury, VA, USA).

### 2.4. Treatments

Docetaxel, a taxoid anti-cancer agent, was purchased from Millipore-Sigma and dissolved in DMSO at a starting concentration of 100 μM. SAHA, a commercially available FDA-approved HDACi, was purchased from Sigma-Aldrich (St. Louis, MO, USA) and dissolved in DMSO at a starting concentration of 100 mM. SBI-I-19, an HDAC inhibitor conjugated with anti-androgen [18], was dissolved in DMSO at a starting concentration of 50 mM. The anti-androgen drugs enzalutamide and bicalutamide were purchased from Selleckchem (Houston, TX, USA) and Sigma-Aldrich, respectively. Anti-androgens were prepared at a stock concentration of 100 mM (enzalutamide) and 10 mM (bicalutamide) in DMSO. The Gα_i_2 inhibitor (compound **14**) was diluted in DMSO at a stock concentration of 100 mM, as previously described [8]. All drugs used in the study were then diluted in the culture media to the final concentrations used in various assays [7,8]. To determine the effects of specific treatments on the levels of Gα_i_2 protein, LNCaP and PC3 cells (10^6^ cells) were cultured overnight in 10 cm^2^ culture dishes. The cells were then treated with anti-androgens and other agents for 24 h and the total cell lysates were collected for Western blot analyses.

### 2.5. Cell Migration Assays

In vitro transwell migration assays were conducted as described previously [7]. Briefly, the outside of the transwell inserts (BD Biosciences, San Jose, CA, USA) were coated with 50 µL of rat tail collagen (50 μg/mL; BD Biosciences, San Jose, CA, USA) overnight at 4 °C. The next day, 50 μL of the aforementioned rat tail collagen was added for 1 h at room temperature into the transwell inserts to coat the inside of the membranes. DU145, PC3 and LNCaP cells were trypsinized and suspended at the appropriate density (3 × 10^5^ cells/insert for DU145 and PC3, and 5 × 10^5^ cells/insert for LNCaP) in MEM or RPMI containing 0.2% BSA (Sigma-Aldrich, St. Louis, MO, USA). Chemoattractant solution, used as the positive control, was made by diluting the epidermal growth factor (EGF) (10 ng/mL) into MEM (PC3 and DU145) or RPMI (LNCaP) supplemented with 0.2% BSA. Media containing 0.2% BSA served as a control. Control and EGF solutions (400 μL) were added into the wells of a 24-well plate. Aliquots of 100 μL of cell suspensions, treated with or without the different compounds at several concentrations, were loaded into the transwell inserts, and the plates were incubated at 37 °C for 5 h (DU145 and PC3) or 24 h (LNCaP). The cells inside the transwell inserts were removed by cotton swabs, and the cleaned inserts were fixed with 350 μL of 4% paraformaldehyde (pH 7.5) for 20 min at room temperature. Cells that had migrated to the outside of the transwell insert membrane were stained with 3 ng/mL of DAPI solution (Thermo Fisher Scientific, Eugene, OR, USA), and images of five non-overlapping fields were captured using an Axiovert 200M, Carl Zeiss (Thornwood, NY, USA) microscope. The number of stained nuclei were determined with automatic counting using image analysis software (ZEN 2012 (blue edition, Version 1.1.1.0); Carl Zeiss Microscopy GmbH, Oberkochen, Germany). The results were expressed as a migration index defined as the average number of cells per field for test substance/the average number of cells per field for the medium control.

### 2.6. Western Blot Analysis

Western blot analyses were performed as described previously [7]. Briefly, cell lysates were mixed with Laemmeli’s buffer (62.5 mM Tris, pH 6.8, 2% sodium dodecyl sulfate, 5% β–mercaptoethanol and 10% glycerol). Samples (30–35 µg proteins) were separated on 10% SDS-PAGE gels and transferred to polyvinylidene difluoride (PVDF) membranes (Millipore Corp., Bedford, MA, USA). After blocking the membranes with 5% fat-free milk in TBST (50 mM Tris, pH 7.5, containing 0.15 M NaCl, and 0.05% Tween 20) for 1 h at room temperature, the membranes were incubated with specific primary antibodies at appropriate dilutions (1:5000 for Gα_i_2; 1:3000 for α-tubulin) overnight at 4 °C. After washing, the blots were incubated with appropriate secondary antibodies. The blots were then developed in Millipore Luminata Forte (EMD Millipore) for 5 min and visualized using a BioRad ChemiDoc Imaging System, according to the manufacturer’s instructions. The density of specific protein bands was determined using ImageJ 1.53 K image analyses software.

### 2.7. Statistical Analysis

All experiments were repeated at least three times using different cell preparations, and two biological replicates were used for each condition. The results are presented as mean ± SEM. One-way ANOVA was employed to assess the significance of differences among various treatment groups (*p* < 0.05) using SigmaPlot 11.0 software.

## 3. Results

### 3.1. Optimization of the First Generation Gα_i_2 Inhibitors

**Scheme 1:**
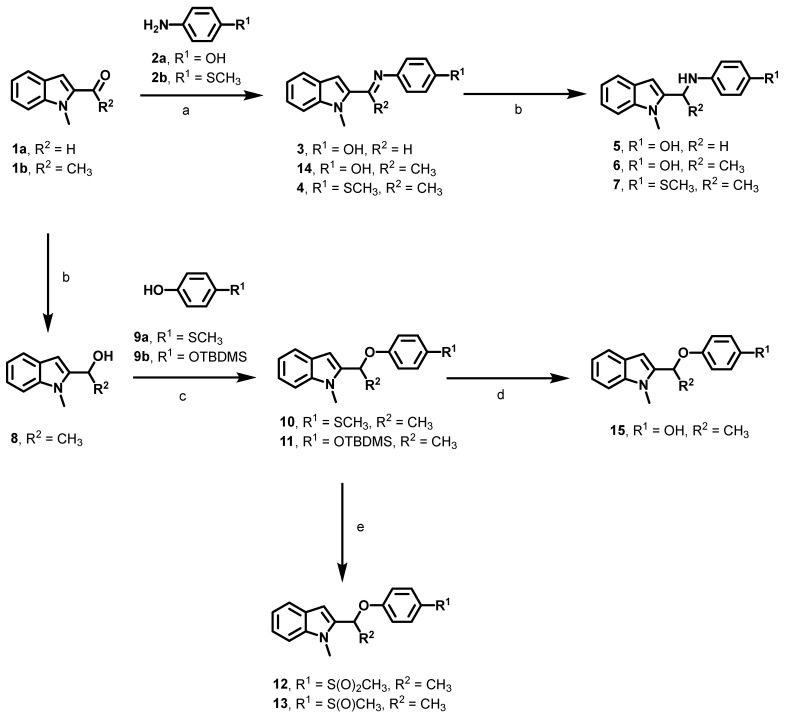
Synthesis of the disclosed Gα_i_2 inhibitors. Reagents and conditions: (a) EtOH, glacial acetic acid, 95 °C, pressure tube; (b) NaBH_4_, MeOH, rt, 45 min; (c) DIAD, PPh_3_, THF, rt, 20–24 h; (d) CsF, MeOH, rt, 2 h; (e) Oxone, iPr_2_NH, CH_3_CN/H_2_O, rt, 1 h.

We have disclosed previously that compound **14** attenuated the EGF-induced migration of prostate cancer cell lines PC3 and DU145 [8]. However, **14** is a phenolic imine, which is sometimes more stable than other imines [26,27] but could still be beleaguered by metabolic liability in in vivo settings. Hence, we first set out to identify analogs that may be devoid of metabolically liable moieties but with improved or comparable potency as **14**. Molecular docking analysis, using Autodock Vina [28] on the structure Gα_i_–GDP (PDB: 2OM2), revealed that **14** adopted a docked pose, suggesting its imine moiety could be replaced with a more stable amino or ether group, while modifications that introduced steric hindrance at the phenolic moiety may not be so well tolerated due to the need to accommodate Mg^2+^ ion at the binding site. Based on this observation, we designed compounds **3**–**7**, **10**, **12**–**13** and **15**, analogs of **14** modified within the aforementioned moieties and c-methyl group. These compounds were facilely synthesized as shown in Scheme 1. Briefly, the reaction of **1a**–**b** with **2a**–**b** and glacial acetic acid in EtOH at 95 °C furnished imines **3**–**4** and the previously disclosed compound **14**. The reduction of **3**–**4** and **14** with NaBH_4_ in MeOH resulted in secondary amines **5**–**7** as racemic mixtures. Similarly, NaBH_4_ reduction of **1b** furnished compound **8**, which was subjected to the Mitsunobu reaction [29] with **9a**–**b** to furnish ether compounds **10**–**11**. Oxidation of the thiomethyl group of **10** by treatment with oxone [30] afforded methylsulfone **12** and methylsulfoxide **13** as racemic and diastereomeric mixtures, respectively. The TBDMS group of **11** was deprotected with CsF to afford compound **15** as a racemic mixture.

### 3.2. Effect of New Compounds on the Migration of Prostate Cancer Cell Lines

**Figure 1:**
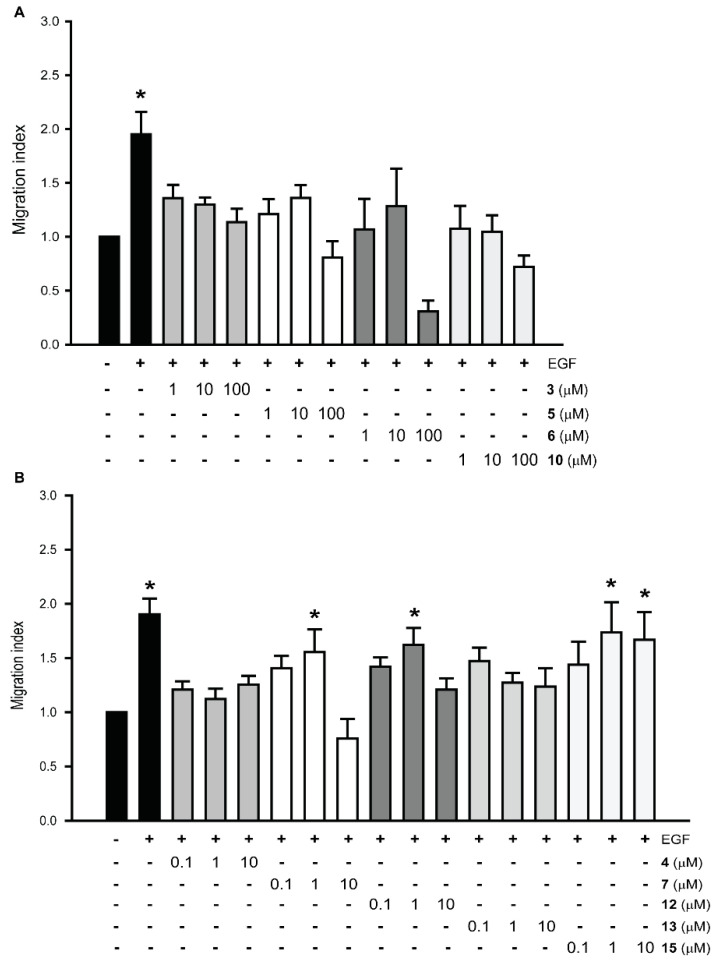
Second-generation compounds differently modulated cell migration in PC3 prostate cancer cell lines. PC3 cells were incubated with (+) or without (−) compounds **3**, **5**, **6** and **10** (**A**), as well as compounds **4**, **7**, **12**–**13** and **15** (**B**) at several concentrations and then subjected to transwell migration assay. EGF (10 ng/mL) was used as positive control. Results are expressed as migration index. Each bar represents mean ± SEM (n = 3). The asterisks on each bar represent significant differences (*p* < 0.05) among various treatment groups, compared to the controls.

**Figure 2:**
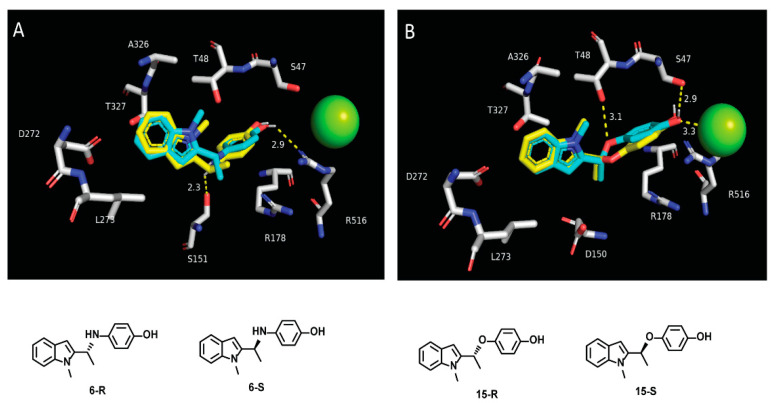
Dock poses of enantiomers of **6** (6-R and 6-S) and **15** (15-R and 15-S) at Gα_i_ active site. (**A**) **6-R** (blue) is accommodated at the Gα_i_ active site with a slightly enhanced binding affinity relative to **6-S** (yellow) (8.6 kcal/mol vs. 8.1 kcal/mol). (**B**) **15-R** (blue) and **15-S** (yellow) bind to Gα_i_ with the same binding affinity (8.0 kcal/mol), which is relatively weaker than that of **6-R**.

We investigated the effects of the newly synthesized compounds on the migratory behavior of PC3 cells in transwell migration assays as described previously [8]. Compounds were tested at a concentration range of 0.1 to 100 μM. The new imine compounds **3** and **4** caused a concentration dependent reduction of PC3 cell migration, but less efficiently relative to **14**. Interestingly, the secondary amines **5**–**7** more efficiently attenuated PC3 migration, with **5** having comparable potency and compounds **6** and **7** being more effective relative to **14**. The ether compounds **10**, **12**, **13** and **15** displayed varying effects on PC3 migration. Compound **10**, an analog in which the amino group for **7** has been switched to an ether group, is less potent compared to **7**. This suggests that the accommodation within the active site of Gα_i_–GDP of the thiomethyl moiety, common to both compounds, is highly dependent on the type of moiety at the para-position connecting to the methylindole group. The methylsulfone **12** and methylsulfoxide **13** are less effective, eliciting reduced anti-migration activities relative to the other compounds (Figure 1). Surprisingly, compound **15**, an ether analog of the potent amine **6**, did not inhibit the migration of PC3 cells at the maximum concentration tested (Figure 1). To potentially obtain insight into the disparity in their Gα_i_ inhibition activities, we performed molecular docking on the enantiomers of **6** (**6-R** and **6-S**) and **15** (**15-R** and **15-S**). We observed that the enantiomeric pair **6-R** and **6-S** bind to Gα_i_ with slightly better binding energies than **14** while adopting similar orientation (Figure 2). Additionally, **6-S** seems to have a slightly improved binding affinity relative to **6-R**. The enantiomeric pair **15-R** and **15-S** are also well accommodated within the Gα_i_ active site with the same binding affinity. However, their binding affinity is slightly weaker than that of **6-R** and **6-S** (Figure 2). This slightly weaker binding affinity may partially explain the disproportionately weaker potency of **15** relative to **6**. Other molecular factors, such as those which led to the attenuated potency that we observed with the amino- to ether-group switch in the **7**/**10** pair, may contribute to the reduced potency of **15**. Finally, most of these compounds have no effects on the viability of several cancer cells. (Supplementary Figure S1A,B).

### 3.3. Evaluation of In Vitro Stability of Representative Compounds

On the strength of their potency, we determined the stability of **6**, **7** and **10** in human and mouse plasma and liver microsomes. We observed that these compounds are stable in the plasma of either species, with a half-life ranging from 27 min to >120 min. In the liver microsomes, the stability of these compounds is highly species-specific, with **6** robustly stable in both species, while **7** and **10** are only stable in the human microsomes (Table 1).

### 3.4. HDACi Induce Cell Migration in LNCaP Prostate Cancer Cell Lines

**Figure 3:**
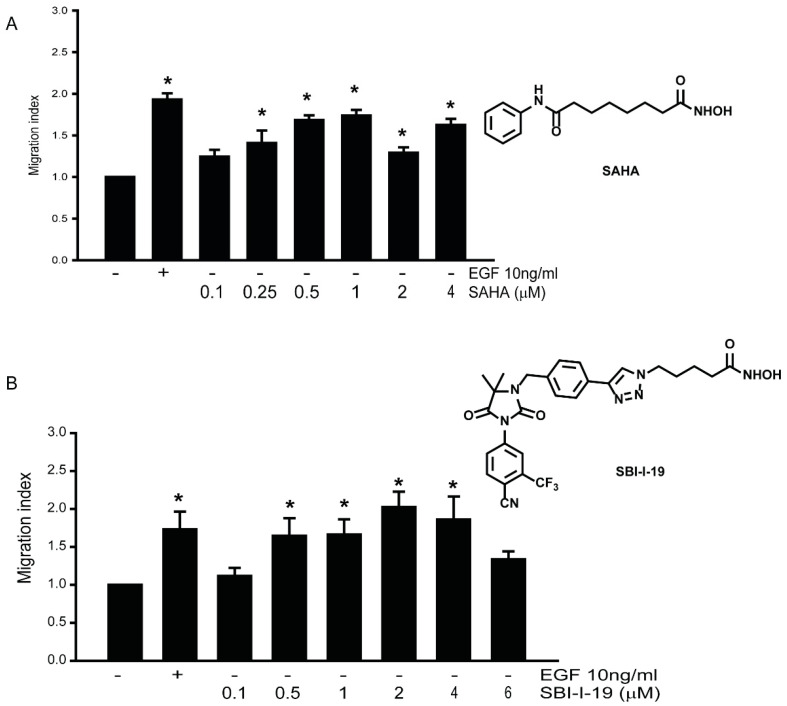
HDACi induced cell migration in LNCaP prostate cancer cell lines. LNCaP cells were incubated with (+) or without (−) SAHA (**A**) or SBI-I-19 (**B**) at several concentrations and then subjected to transwell migration assay. EGF (10 ng/mL) was used as positive control. Results are expressed as migration index. Each bar represents mean ± SEM (n = 3). The asterisks (*) on each bar represent significant differences (*p* < 0.05) among various treatment groups, compared to the controls. Chemical structures of SAHA and SBI-I-19 are depicted with respective bar diagrams.

To determine the effects of HDACi on prostate cancer cell migration, we performed migration assays in AR-positive LNCaP cells using several concentrations of SAHA (0.1, 0.25, 0.5, 1, 2 and 4 μM), a commercially available HDACi. As shown in Figure 3A, treatments with SAHA induced a significant dose-dependent increase in cell migration. Maximum induction of cell migration was observed at 0.5 and 1 µM doses of SAHA. We have previously developed a very potent HDACi inhibitor (SBI-I-19), coupled with an enzalutamide-like anti-androgen moiety, which selectively targets androgen sensitive prostate cancer cells for its cytotoxic effects [18]. We determined the possible effects of different concentrations of SBI-I-19 on cell migration in LNCaP cells. As shown in Figure 3B, SBI-I-19 also induced a significant dose-dependent increase in cell migration, when compared to the untreated cells, with maximum effects observed at 1 and 2 μM.

### 3.5. Anti-Androgens Induce Cell Migration in AR-Positive LNCaP Prostate Cancer Cells

**Figure 4:**
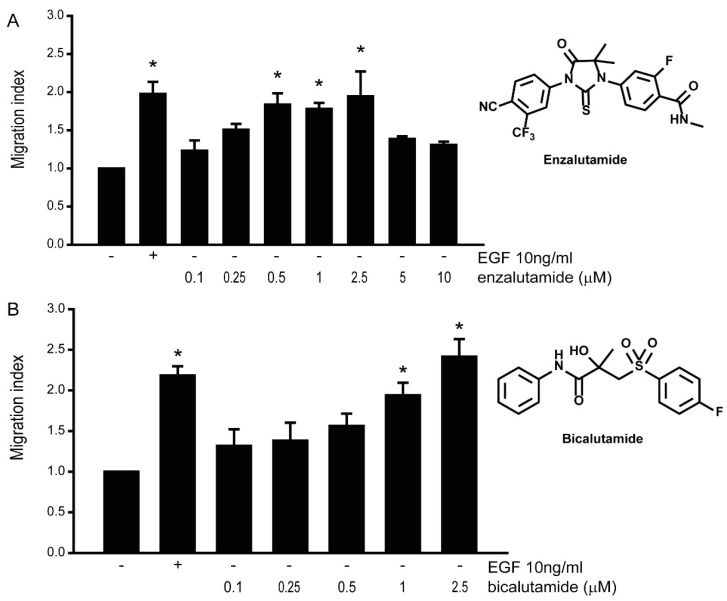
Anti-androgens induced cell migration in LNCaP prostate cancer cell lines. LNCaP cells were incubated with (+) or without (−) enzalutamide (**A**) or bicalutamide (**B**) at several concentrations and then subjected to transwell migration assays. EGF (10 ng/mL) was used as positive control. Results are expressed as migration index. Each bar represents mean ± SEM (n = 3). The asterisks (*) on each bar represent significant differences (*p* < 0.05) among various treatment groups, compared to the controls. Chemical structures of enzalutamide and bicalutamide are depicted with respective bar diagrams.

Since SBI-I-19 was equipped with an anti-androgen moiety inspired by enzalutamide, this anti-androgen moiety could contribute to the observed effects of SBI-I-19 on cell migration. To investigate this possibility, we treated LNCaP cells with different concentrations of two FDA-approved anti-androgen drugs (enzalutamide and bicalutamide). To our surprise, as shown in Figure 4A,B, both anti-androgens induced a dose-dependent increase in cell migration in LNCaP cells. The maximum effects of both compounds were observed at a dose of 2.5 μM.

To determine whether the chemotherapeutic drugs affected cell viability and proliferation, we treated LNCaP cells with different concentrations of the drugs and performed MTS assays and proliferation assays using Incucyte SX5 system (Supplementary Figure S2). The drugs did not affect significantly the proliferation of LNCaP cells within 24 h.

### 3.6. Effects of Chemotherapy on Cancer Cell Migration in AR-Negative DU145 and PC3 Cells

**Figure 5:**
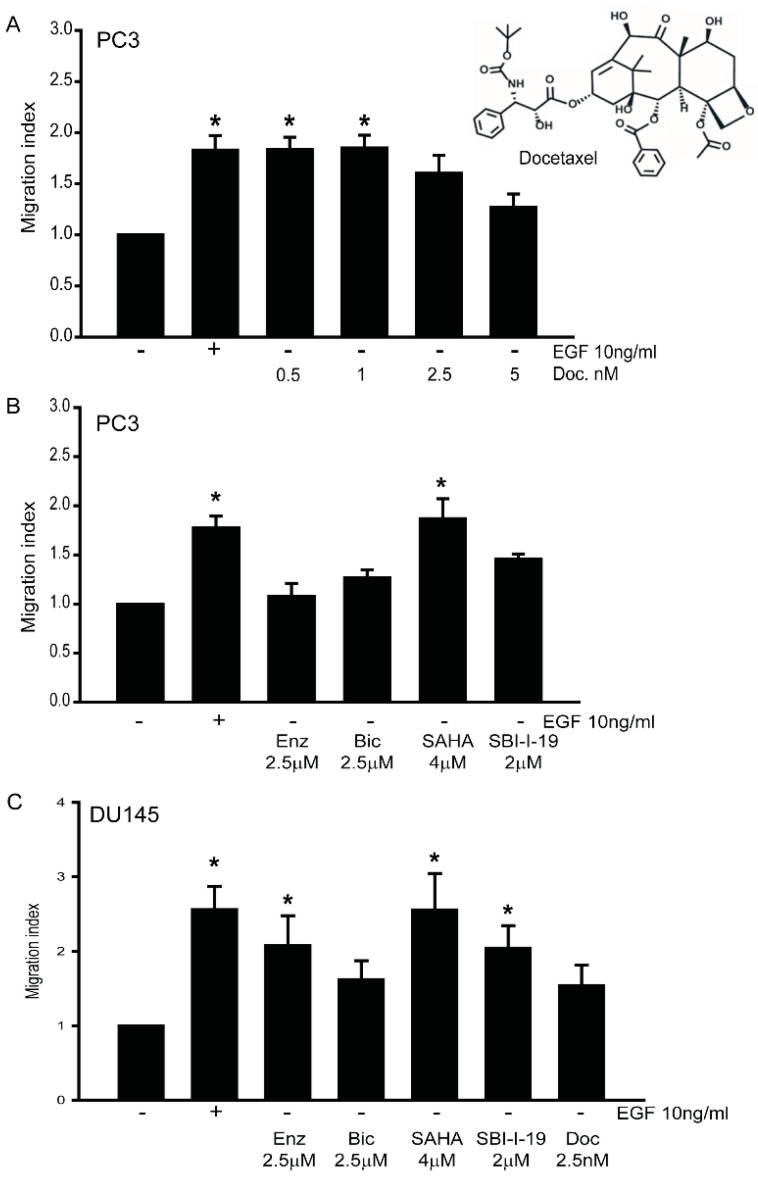
Docetaxel, SAHA and anti-androgen effects on cell migration in PC3 and DU145 prostate cancer cell lines. PC3 (**A**,**B**) and DU145 (**C**) cells were incubated with (+) or without (−) different agents and then subjected to transwell migration assays. EGF (10 ng/mL) was used as positive control. Results are expressed as migration index. Each bar represents mean ± SEM (n = 3). The asterisks (*) on each bar represent significant differences (*p* < 0.05) among various treatment groups, compared to the controls. Chemical structure of docetaxel is depicted with respective bar diagrams.

To determine whether the induction of cell migration by AR-antagonists and HDACi was limited to only AR-positive cells, we determined the effects of anti-androgens, HDACi and docetaxel on cell migration on AR-negative DU145 and PC3 cells. Both anti-androgens did not have any effect on cell migration in PC3 cells (Figure 5B). On the other hand, treatments with HDACi (SAHA) or docetaxel, a chemotherapeutic drug commonly utilized to treat advanced castration-resistant metastatic prostate cancers, induced an increase in cell migration in these cells (Figure 5A,B). In DU145 cells, treatments with enzalutamide and HDACi (SAHA and SBI-I-19) increased cell migration as well (Figure 5C).

### 3.7. Anti-Androgens Upregulate the Expression of Gα_i_2 Protein in LNCaP Cells

**Figure 6:**
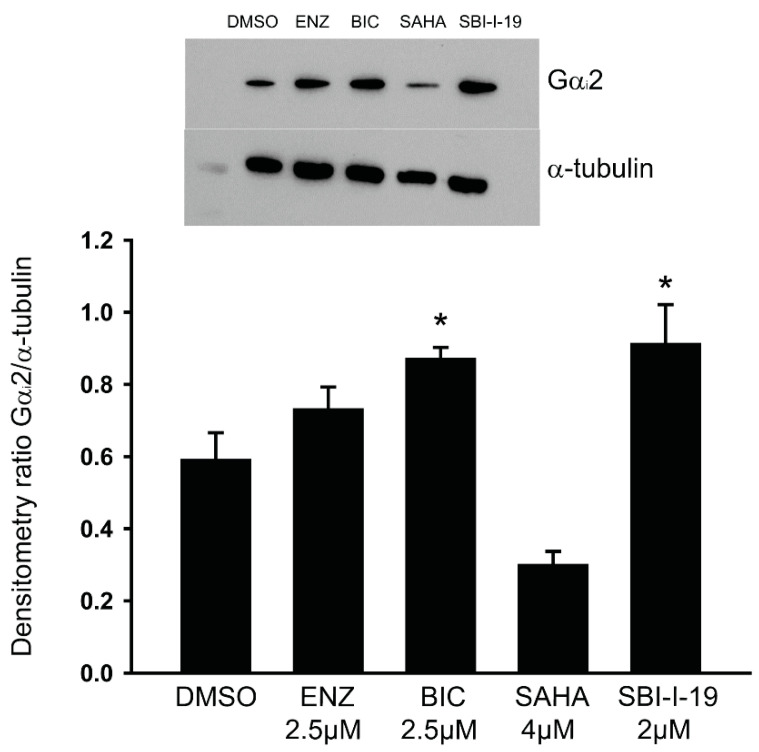
Anti-androgens induced an increase in the levels of Gα_i_2 protein in LNCaP cell lines. LNCaP cells were incubated for 24 h with enzalutamide (2.5 μM), bicalutamide (2.5 μM), SAHA (4 μM) and SBI-I-19 (2 μM) and then subjected to Western blot analysis to evaluate the expression of Gα_i_2 protein. DMSO was used as control. Results are expressed as ratio between Gα_i_2 band densitometry/α-tubulin band densitometry. Each bar represents mean ± SEM (n = 3). The asterisks (*) on each bar represent significant differences (*p* < 0.05) against the control (DMSO). Full blots are shown in the Supplementary Information (Figure S3).

Previously, we have shown the essential role of Gα_i_2 protein in prostate cancer cell migration [7]. To determine whether the HDACi and anti-androgens regulate the expression of Gα_i_2 protein, we treated LNCaP cells with anti-androgens and HDACi for 24 h, using the most effective concentrations for each drug, and determined the levels of Gα_i_2 protein by Western blotting. As shown in Figure 6, treatments with enzalutamide (2.5 μM), bicalutamide (2.5 μM) and SBI-I-19 (2 μM) resulted in increased levels of Gα_i_2 protein in LNCaP cells. On the other hand, HDACi SAHA induced a reduction in the levels of Gα_i_2 protein. However, this reduction was not sufficient to affect cell migration. In PC3 cells, treatment with HDACi, anti-androgens or docetaxel (1 nM), had no effect on the levels of Gα_i_2 protein.

### 3.8. Gα_i_2 Inhibitors Block the Effects of the Chemotherapeutic Drugs on Prostate Cancer Cell Migration

**Figure 7:**
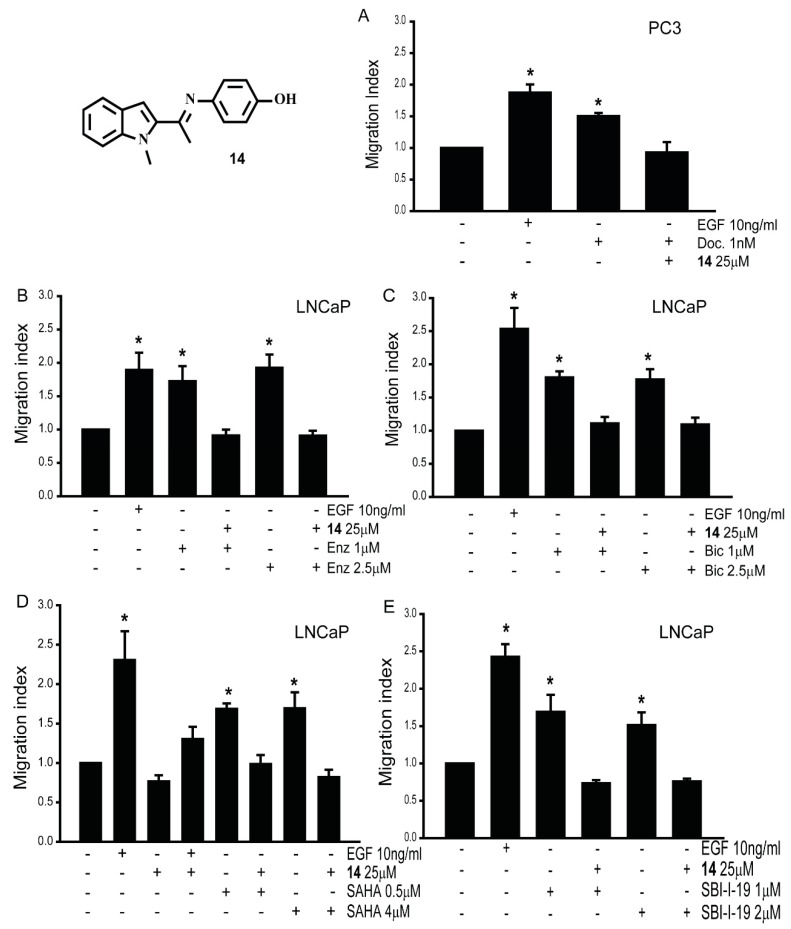
Combination therapies with chemotherapeutic drugs and Gα_i_2 inhibitors decreased prostate cancer cell migration. Prostate cancer cells were incubated with (+) or without (−) Docetaxel ((**A**), PC3), enzalutamide ((**B**), LNCaP), bicalutamide ((**C**), LNCaP), SAHA ((**D**), LNCaP) and SBI-I-19 ((**E**), LNCaP) at appropriate concentrations, with (+) or without (−) compound **14** (25 μM), and then subjected to transwell migration assay in the presence or absence of EGF (10 ng/mL). Results are expressed as migration index. The chemical structure of compound **14** is depicted in the left top corner. Each bar represents mean ± SEM (n = 3). The asterisks (*) on each bar represent significant differences (*p* < 0.05) among various treatment groups, compared to the controls.

We have recently developed first-generation small molecule inhibitors of Gα_i_2 protein activation which block cell migration and invasion in several cancer cell types [8]. In this study, we first used one of the most effective Gα_i_2 inhibitors (compound **14**, at 25 μM) in combination with the above chemotherapeutic drugs. As shown in Figure 7, simultaneous treatment with Gα_i_2 inhibitors blocked the stimulatory effects of docetaxel in PC3 cells (Figure 7A), and HDACi and anti-androgen effects on cell migration in LNCaP cells (Figure 7B–E).

**Figure 8:**
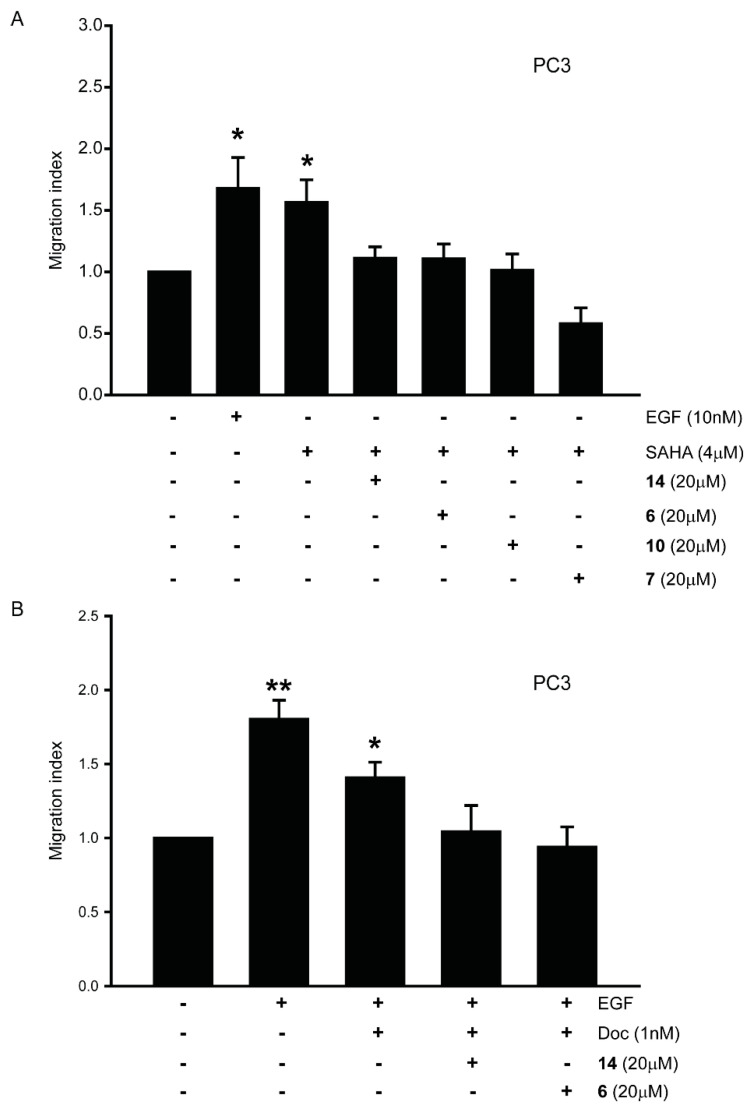
Combination therapies with SAHA or docetaxel and the second-generation Gα_i_2 inhibitors decreased PC3 cell migration. PC3 cells were incubated with (+) or without (−) SAHA (**A**) or docetaxel (**B**) at appropriate concentrations, with (+) or without (−) compound **14** (20 μM), and the second-generation Gα_i_2 inhibitors (compounds **6**, **7**, **10**, at 20 μM) and then subjected to transwell migration assay in the presence or absence of EGF (10 ng/mL). Results are expressed as migration index. Each bar represents mean ± SEM (n = 3). The asterisks on each bar represent significant differences (*p* < 0.05) among various treatment groups, compared to the controls (** significant against all; * significant against the controls).

Subsequently, we used some of the most effective second-generation Gα_i_2 inhibitors (compounds **6**, **7** and **10**, at 20 μM), in combination with either SAHA or docetaxel, and evaluated the migratory capability of PC3 cells. As shown in Figure 8, combination therapies with SAHA or docetaxel and the second-generation Gα_i_2 inhibitors decreased significantly PC3 cell migration.

## 4. Discussion

We have identified potent, metabolically stable Gα_i_2 inhibitors, which we validated as novel inhibitors of intrinsic and EGF-stimulated migration of cancer cells. Other novel findings in this study show that anti-androgens and chemotherapeutic agents (docetaxel and HDACi) induce migratory behavior in prostate cancer cells, and these effects on cell migration can be blocked by simultaneous treatment with small molecule inhibitors of Gα_i_2 protein.

Early stage prostate cancers are localized in the prostate gland and are treatable by surgery and radiation therapy; the prognosis in these patients is very good. However, prostate cancers in later stages of the disease metastasize to other tissues and bone, reducing drastically the overall survival of the patients [31]. The current treatment option available for hormone-sensitive prostate cancers is androgen deprivation therapy (ADT), based on either inhibition of androgen biosynthesis or treatment with AR antagonists. In this study, we observed that two of the most commonly used AR-antagonists, enzalutamide and bicalutamide, caused significant induction of cell migration in AR-positive prostate cancer cells, indicating that treatment with these drugs may lead to unintended consequences resulting in the development or aggravation of metastatic disease. Interestingly, enzalutamide induced cancer cell migration in DU145 cells, which are AR-null prostate cancer cells. One possible explanation could be due to the fact that AR-negative DU145 prostate cancer cells have been shown to respond to enzalutamide treatments through the glucocorticoid receptor (GR), encoded by *NR3C1* gene [32].

Depending on the cancer type and the stage of the disease, surgery followed by chemotherapy is the most common strategy utilized to treat cancer patients that could prove effective and curative [33]. However, the development of distant metastases is quite common in cancer patients despite achieving complete control of the primary tumor [34,35,36]. Several recent studies have shown that chemotherapeutic agents can promote cancer cell migration and participate in the development of metastatic disease [17,37,38]. Due to the broad use of chemotherapeutic agents in cancer treatments, it is critical to investigate their metastasis-promoting activity [39]. We observed in this study that treatment with HDACi and docetaxel resulted in increased migratory behavior in both AR-positive and AR-negative prostate cancer cells, indicating that these effects on cell migration in prostate cancer cells are not limited to only anti-androgens. The mechanisms involved in these effects of various chemotherapies are not well understood. One of the possible events triggering chemotherapy-induced metastases can be achieved by modulating non-cancer host cells and creating a favorable microenvironment for cancer cell dissemination [40,41,42,43]. Additionally, the cell cytotoxicity of the chemotherapeutic agents and/or hypoxia could potentially activate “escape” mechanisms, leading to cancer cell dissemination and metastases [44,45,46]. Whether these escape mechanisms are involved in the induction of epithelial to mesenchymal transformation (EMT) and secretion of MMPs and other enzymes, which lead to increased migratory and invasive behavior in these cells, needs to be determined in future studies. However, these in vitro effects of anti-androgens and other agents (docetaxel and HDACi) were observed at very low doses, which did not exert any cytotoxic effects under the same experimental conditions (Supplementary Figure S2). Therefore, the clinical relevance of these findings remains to be determined as the treatments with these agents in cancer patients are normally carried out at much higher cytotoxic doses. In our earlier studies, we have shown that Gα_i_2 protein plays an essential role in cell migration and invasion in prostate and other cancer cells, and these effects of Gα_i_2 protein are exerted at two distinct levels. One of these effects is dependent on the activation of GPCR signaling by specific ligands (such as SDF1α and PGE2), while the second effect is independent of GPCR signaling and is exerted at the level of lamellipodia formation in response to the activation of multiple upstream pathways [7]. In addition, we have also developed small molecule inhibitors of Gα_i_2 which blocked the migratory behavior of prostate and other cancer cells induced by various ligands [7,8]. In the present study, we observed that treatment with anti-androgens (enzalutamide and bicalutamide) induced an increase in intracellular levels of Gα_i_2 protein in AR-positive LNCaP cells, but not in AR-negative PC3 cells, indicating that effects of anti-androgens on Gα_i_2 levels may be mediated by AR. On the other hand, HDACi (SAHA) caused a reduction in Gα_i_2 in LNCaP cells. However, this reduction did not cause complete elimination of the protein. Therefore, there is enough Gα_i_2 to sustain cell migration. In PC3 cells, which have a very high expression of Gα_i_2, HDACi, anti-androgens or docetaxel (1 nM) did not induce further expression of the protein. Increased Gα_i_2 levels may, in turn, mediate increased cell migration, which also suggests a possible role of AR in the regulation of cell migration. Additional studies are needed to explore this possibility. On the other hand, HDACi and docetaxel had no effect on the expression levels of Gα_i_2 protein, indicating that the effects of these agents are distinct from anti-androgens. However, the essential role of Gα_i_2 protein in cell migration was confirmed since inhibition of its activity by small molecule inhibitors resulted in attenuation of cell migration induced by all chemotherapeutic agents.

Currently, very few metastasis-specific therapeutic targets have been identified, and effective prevention and suppression of metastatic disease is still an elusive goal [36].

## 5. Conclusions

Due to the essential role of Gα_i_2 protein in cell migration and the ability of its inhibitors to block the effects of many different ligands on cell motility, Gα_i_2 inhibitors could be promising anti-metastatic drug candidates. These findings also suggest that combination therapy with Gα_i_2 inhibitors along with anti-androgens and/or other chemotherapeutic agents may stunt the possible negative effects of these treatments in the development of metastatic disease.

## Figures and Tables

**Table 1:** Stability of compounds **6**, **7** and **10** in human and mouse plasma and liver microsomes.

Compound	Plasma (t_1/2_ = Min)		Microsome (t_1/2_ = Min)	
	Human	Mouse	Human	Mouse
**6**	>120	27	118	47
**7**	>120	>120	45	<10
**10**	>120	>120	35	<10

## Data Availability

The data presented in this article are available.

## Supplementary Materials

The following supporting information can be downloaded at: https://www.mdpi.com/article/10.3390/cancers16020296/s1. Figure S1. Effects of compounds **3**–**7**, **10**, **12**, **13** and **15** on the viability of several cells; Figure S2. Cell viability and proliferation of LNCaP cells after treatments with chemotherapeutic drugs; Figure S3. Western blot full blots; Figure S4. Cell viability assay of PC3 cells after 72 h treatments with Gα_i_2 inhibitors and LNCaP cells after 24 h treatments with HDACi combined with compound **14**, lead Gα_i_2 inhibitor; Chemistry information for the synthesis of the compounds.
