# Supplementary material for: Gαi2 Protein Inhibition Blocks Chemotherapy- and Anti-Androgen-Induced Prostate Cancer Cell Migration
Source: Cancers (Basel). 2024 Jan 10;16(2):296. doi: 10.3390/cancers16020296 (PMC10813862; doi:10.3390/cancers16020296)
Supplement: Supplementary file 1 [file cancers-16-00296-s001.zip › cancers-2769377-supplementary.pdf]

Supplementary information:

Gαi2 protein inhibition blocks chemotherapy- and antiandrogen-induce prostate cancer cells migration.

Silvia Caggia[1], Alexis Johnston[2], Dipak T. Walunj[2], Aanya R. Moore[1], Benjamin H. Peer[2], Ravyn W. Everett[1], Adegboyega K. Oyelere[2, 3*], Shafiq A. Khan[1*]

[1]Center for Cancer Research and Therapeutic Development, Clark Atlanta University, Atlanta, 30314, GA, USA; [2]School of Chemistry and Biochemistry and [3]Parker H. Petit Institute for Bioengineering and Bioscience, Georgia Institute of Technology, Atlanta, 30318, GA, USA

***Correspondence:**

Shafiq A. Khan, Ph.D.

Center for Cancer Research and Therapeutic Development

Clark Atlanta University, 223 James P. Brawley Dr, SW, Atlanta, GA 30314

E-mail: skhan@cau.edu

Adegboyega K. Oyelere, Ph.D.

School of Chemistry and Biochemistry and Parker H. Petit Institute for Bioengineering and Bioscience, Georgia Institute of Technology

901 Atlantic Drive, Atlanta, GA 30332-0400

E-mail address: aoyelere@gatech.edu

Supplementary Materials

Cell cytotoxicity data

Figure S1A. Effects of compounds **3-7**, **10**, **12**, **13** and **15** on the viability of LNCaP, MDA-MD-231, MCF-7 and Vero cells. The cells are incubated with the drugs for 72 h and viability evaluated with MTS. NI = IC_{50} greater than 100 µM. n=3

Compound	LNCaP (µM)	MDA-MB-231 (µM)	MCF-7 (µM)	Vero (µM)
**3**	NI	NI	NI	NI
**4**	NI	NI	NI	NI
**5**	NI	NI	NI	NI
**6**	86.8 ± 10.8	27.3 ± 4.8	75.7 ± 5.3	32.4 ± 1.6
**7**	43.4 ± 3.9	NI	NI	NI
**10**	NI	NI	NI	NI
**12**	NI	NI	NI	NI
**13**	NI	NI	NI	NI
**15**	NI	NI	NI	NI

Figure S1B. Dose-response curves showing the effects of compounds **3-7**, **10**, **12**, **13** and **15** on the viability of LNCaP, MDA-MD-231, MCF-7 and Vero cells..

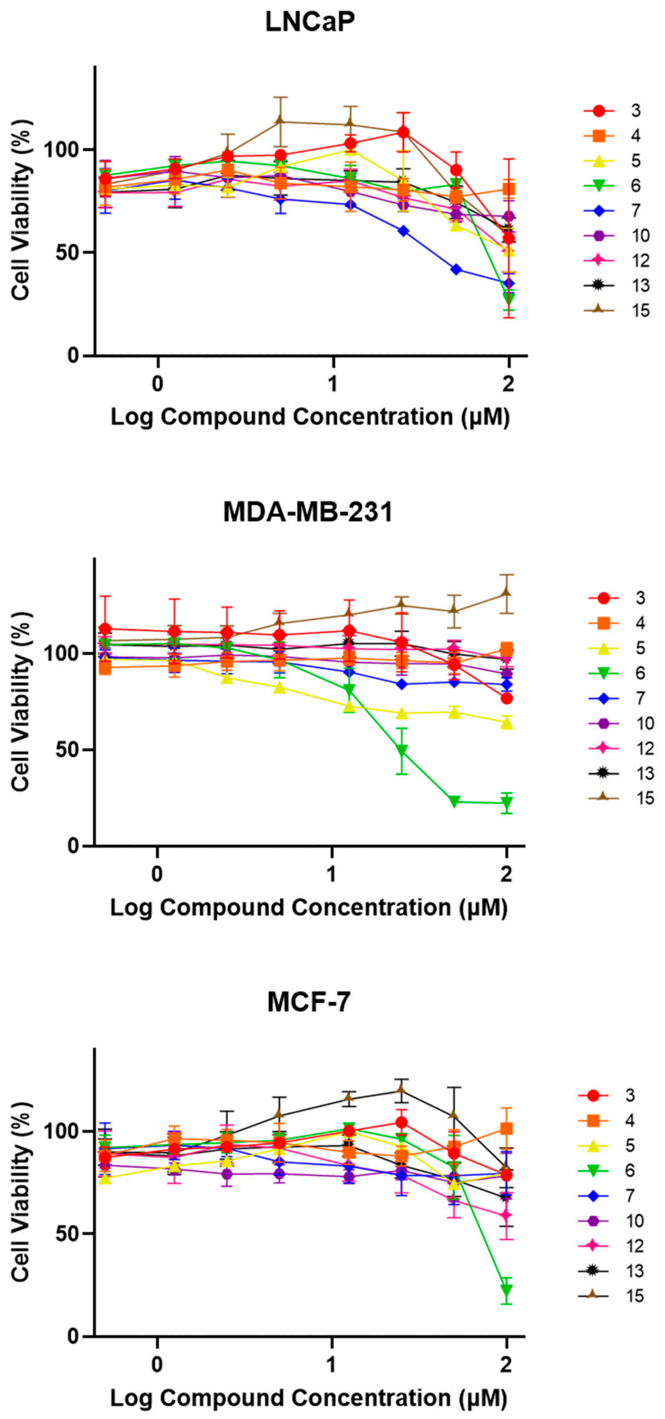

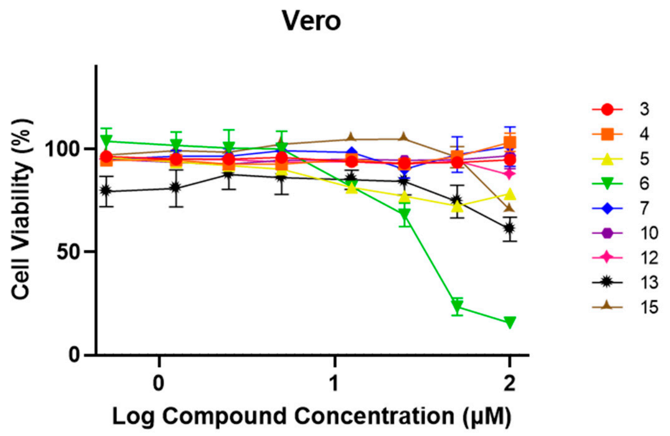
Vero
Cell Viability (%)
100
50
0
0
1
2
Log Compound Concentration (μM)
3
4
5
6
7
10
12
13
15

Figure S2. Cell viability and proliferation of LNCaP cells after treatments with chemotherapeutic drugs.

Supplementary Figure 2

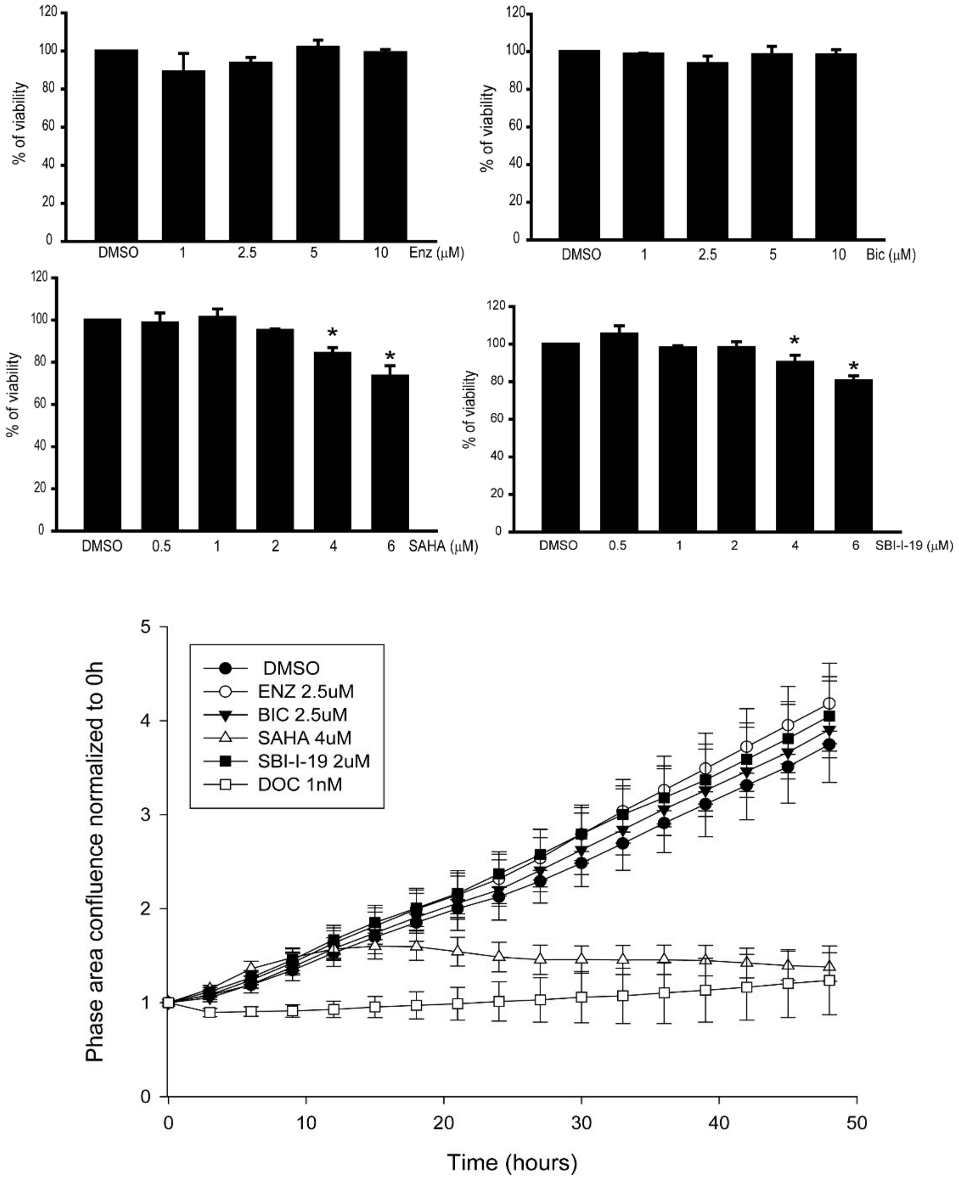

Figure S3. Western blot full blots.

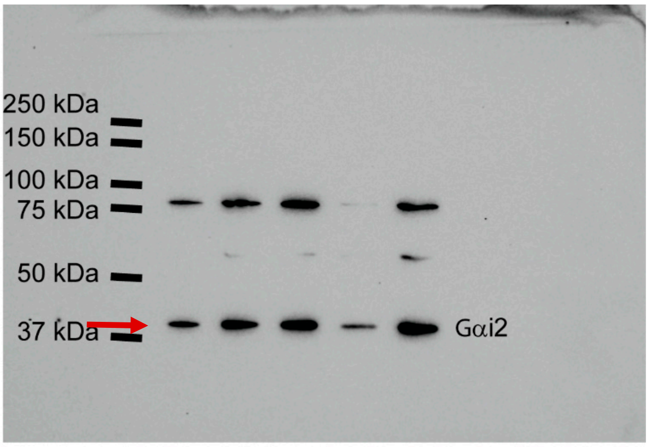

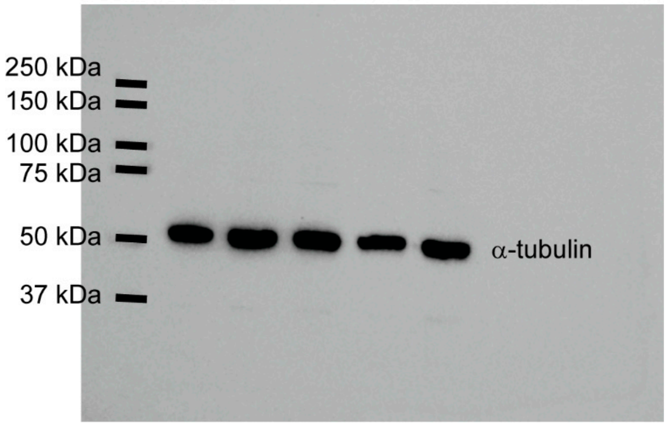

Figure S4. (A) Cell viability assay of PC3 cells after 72h treatments with Gαi2 inhibitors. (B) Cell viability assay of LNCaP cells after 24h treatents with HDACi combined with compound **14**, lead Gαi2 inhibitor.

Supplementary Figure 4

A

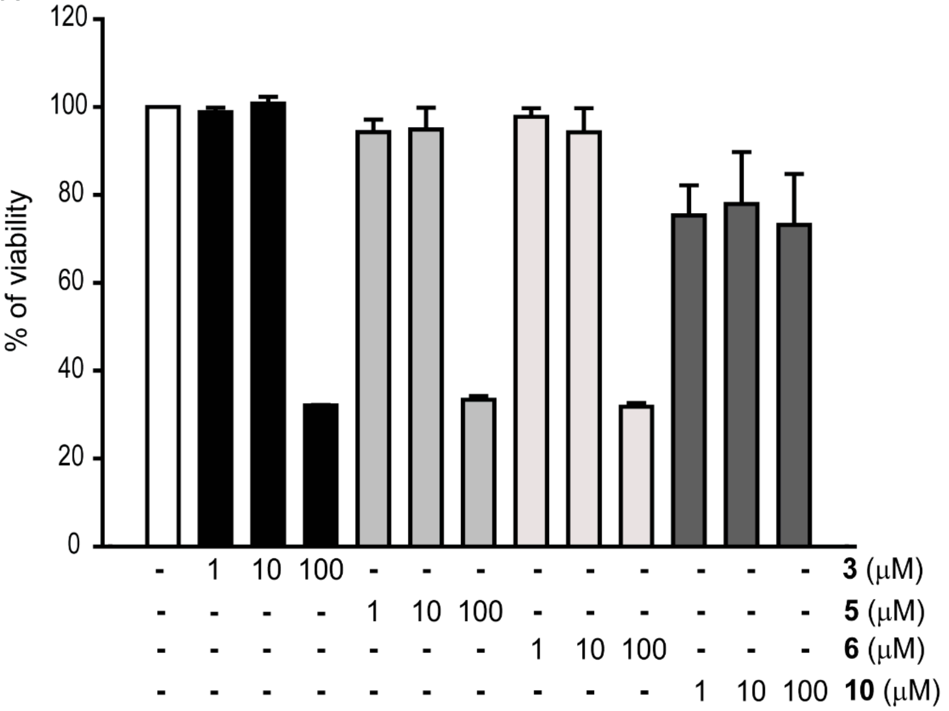

B

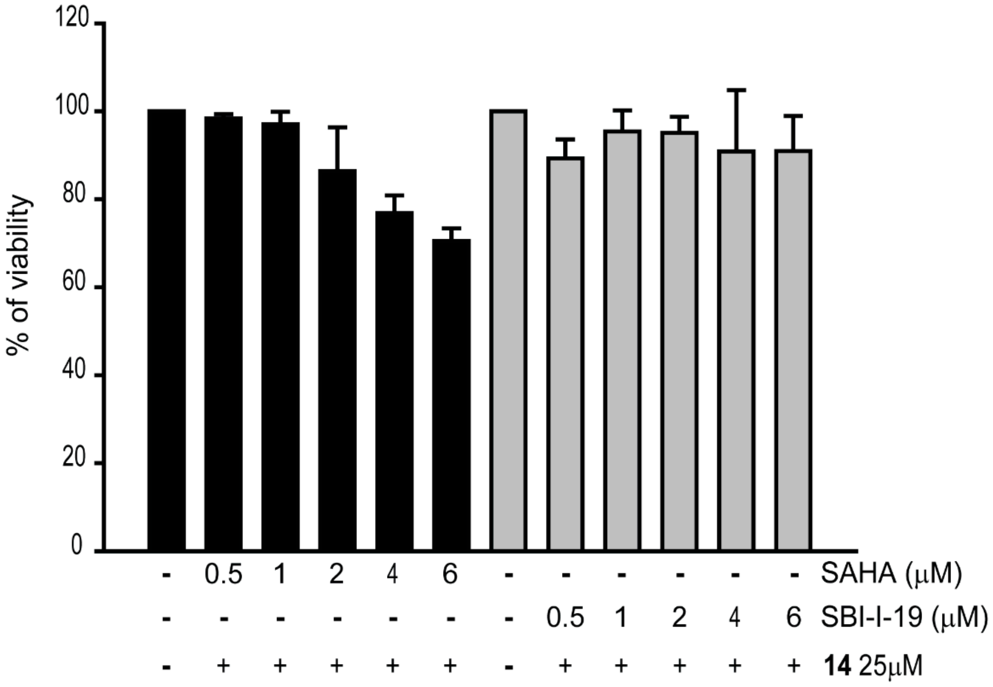

Chemistry

Representative procedure for the synthesis of ketimines.

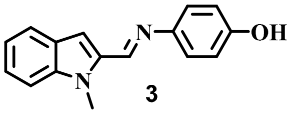

3

Synthesis of (E)-4-(((1-methyl-1H-indol-2-yl)methylene)amino)phenol (**3**): A mixture of 1-methyl-1H-indole-2-carbaldehyde **1a** (483 mg, 3.14 mmol), 4-aminophenol **2a** (220 mg, 2.02 mmol) and glacial acetic acid (0.2 mL) in dry EtOH (10 mL) was heated in a pressure tube at 95°C for 1.5 h during which TLC revealed a quantitative consumption of **2a**. The solvent was evaporated and the reddish paste was co-evaporated with CH_2Cl_2 (3 x 15 mL). The residue was suspended in Et_2O/CH_2Cl_2 6:4 (20 mL) and kept in the refrigerator overnight. The suspension was filtered and the residue was copiously washed with ambient temperature Et_2O (ca. 60 mL). The brick-red precipitate was air-dried and then completely dried *in vacuo*. The filtrate was evaporated off and the reddish paste was suspended in Et_2O/CH_2Cl_2 7:3 (20 mL) and kept in the refrigerator overnight. The suspension was filtered to give a second crop of the desired compound **3**. Combined yield = 270 mg (1st crop) + 30 mg (2nd crop) = 300 mg (59 %). ^{1}H NMR (700 MHz, DMSO-*d6*) δ 9.52 (s, 1H), 8.71 (s, 1H), 7.64 (d, *J* = 8.4 Hz, 1H), 7.52 (d, *J* = 7.6 Hz, 1H), 7.28 (t, *J* = 7.6 Hz, 1H), 7.24 (d, *J* = 8.5 Hz, 2H), 7.09 (t, *J* = 7.5 Hz, 1H), 6.82 (d, *J* = 8.9 Hz, 2H), 4.17 (s, 3H). ^{13}C NMR (176 MHz, DMSO-*d6*) δ 156.5, 149.6, 143.0, 139.9, 136.0, 126.9, 124.2, 122.6, 121.7, 120.3, 116.0, 110.5, 109.7, 31.8. HRMS (EI) m/z Calcd. for $C_{16}H_{15}ON_2$ $[M + H]^+$: 251.1179, found 251.1177.

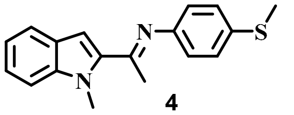

4

(E)-1-(1-methyl-1H-indol-2-yl)-N-(4-(methylthio)phenyl)ethan-1-imine (**4**): The reaction of 1-(1-methyl-1H-indol-2-yl)ethan-1-one **1b** (154 mg, 0.89 mmol), 4-(methylthio)aniline **2b** (0.5 mL, 3.9 mmol) and glacial acetic acid (0.1 mL) (without EtOH) in a pressure tube under reflux (105 – 110 °C) within 24 h, followed by purification on silica gel column chromatography eluting with EtOAc/Hexanes 1:8, gave **4** as light-yellow solid. Yield = 146 mg (56 %). ^{1}H NMR (700 MHz, $CDCl_3$) δ 7.66 (d, *J* = 8.9 Hz, 1H), 7.39 (d, *J* = 0.9 Hz, 1H), 7.38 (d, *J* = 1.0 Hz, 1H), 7.31 (d, d, *J* = 8.7 Hz, 2H), 7.14 (t, *J* = 8.0 Hz, 1H), 7.04 (d, *J* = 0.9 Hz, 1H), 6.77 (d, *J* = 8.7 Hz, 2H), 4.15 (s, 3H), 2.51 (s, 3H), 2.30 (s, 3H). ^{13}C NMR (176 MHz, $CDCl_3$) δ 160.3, 148.8, 140.0, 137.3, 132.2, 128.6, 126.4, 124.1, 121.6, 120.3, 120.1, 110.0, 107.7, 33.0, 18.7, 17.1. HRMS (EI) m/z Calcd. for $C_{18}H_{18}SN_2$ $[M + H]^+$: 295.1263, found 295.1261.

Representative procedure for the reduction of ketimines with $NaBH_4$.

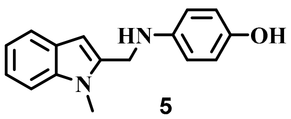

5

Synthesis of 4-(((1-methyl-1H-indol-2-yl)methyl)amino)phenol (**5**): To a solution of **3** (120 mg, 0.48 mmol) in MeOH (10 mL) was added $NaBH_4$ (75 mg, 1.92 mmol) upon which a rapid bubbling occurred. Stirring continued at rt for 45 min during which TLC revealed a quantitative conversion to a lower rf product. Water (30 mL) was added and the milky suspension was extracted with CH_2Cl_2 (4 x 20 mL). The combined organic layer was washed with brine (30 mL) and dried over Na_2SO_4. The solvent was evaporated off to give **5** as a brownish-white solid. Yield = 113 mg (93 %). ^{1}H NMR (400 MHz, $CDCl_3$) δ 7.57 (d, *J* = 7.9 Hz, 1H), 7.31 (d, *J* = 8.2 Hz, 1H), 7.24 – 7.19 (m, 1H), 7.12 – 7.07 (m, 1H), 6.71 (d, *J* = 8.7 Hz, 2H), 6.62 (d, *J* = 8.9 Hz, 2H), 6.45 (s, 1H), 4.36 (s, 2H), 3.74 (s, 3H). ^{13}C NMR (176 MHz, $CDCl_3$) δ 149.3, 149.3, 141.3, 137.8, 137.7, 127.4, 121.3, 120.3, 120.2, 119.3, 116.0, 115.0, 109.0, 100.4, 41.8, 29.6. MS $[M+H]^+$: 253.3

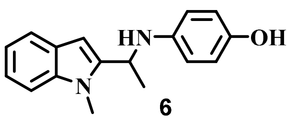

6

4-((1-(1-methyl-1H-indol-2-yl)ethyl)amino)phenol (**6**): The reduction of **14** (110 mg, 0.42 mmol) with $NaBH_4$ (120 mg, 3.2 mmol) in MeOH (15 mL), as described for compound **5**, furnished the desired compound **6** as a reddish-white solid. Yield = 110 mg (99 %). ^{1}H NMR (400 MHz, $CDCl_3$) δ 7.56 (dd, *J* = 7.9, 1.0 Hz, 1H), 7.32 – 7.28 (m, 1H), 7.20 (t, *J* = 7.6 Hz, 1H), 7.08 (t, *J* = 7.9 Hz, 1H), 6.67 (d, *J* = 8.5 Hz, 2H), 6.54 (d, *J* = 8.6 Hz, 2H), 6.46 (t, *J* = 0.8 Hz, 1H), 4.69 (q, *J* = 6.5 Hz, 1H), 3.73 (s, 3H), 1.62 (d, *J* = 6.5 Hz, 3H). 13C NMR (176 MHz, $CDCl_3$) δ 148.0, 142.8, 141.2, 138.0, 127.4, 121.5, 120.6, 119.6, 116.4, 115.1, 109.1, 98.8, 47.1, 30.0, 20.5. MS $[M+H]^+$: 267.1.

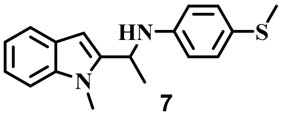

7

N-(1-(1-methyl-1H-indol-2-yl)ethyl)-4-(methylthio)aniline (**7**): The reduction of **4** (100 mg, 0.34 mmol) with $NaBH_4$ (120 mg, 3.2 mmol) in MeOH (10 mL) and CH_2Cl_2 (5 mL), as described for compound **5**, after purification by prep-TLC eluting with Hexanes/EtOAc 7:1, furnished the desired compound **7** as a solid. Yield = 55 mg (55 %). ^{1}H NMR (700 MHz, $CDCl_3$) δ 7.57 (d, *J* = 9.9 Hz, 1H), 7.30 (s, 1H), 7.21 (d, *J* = 10.1 Hz, 3H), 7.10 (d, *J* = 7.0 Hz, 1H), 6.59 (d, *J* = 8.7 Hz, 2H), 6.48 (s,1H), 4.78 (q, *J* = 6.6 Hz, 1H), 3.73 (s, 3H), 2.41 (s, 3H), 1.66 (d, *J* = 6.6 Hz, 3H). ^{13}C NMR (176 MHz, $CDCl_3$) δ 145.6, 142.0, 137.9, 131.5, 127.3, 124.8, 121.6, 120.5, 119.5, 113.7, 109.0, 98.9, 45.9, 29.8, 20.2, 19.0. MS $[M+H]^+$: 297.2.

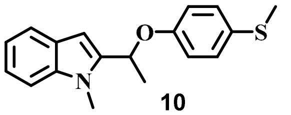

10

1-methyl-2-(1-(4-(methylthio)phenoxy)ethyl)-1H-indole (**10**): Compound **1b** (556 mg, 3.2 mmol) was treated with $NaBH_4$ (488 mg, 12.84 mmol) in MeOH (30 mL) at rt for 45 min during which TLC revealed a quantitative formation of a lower rf product (rf = 0.24, EtOAc/Hexanes 1:5). The reaction was worked up as described for the synthesis of **5** and the resulting crude 1-(1-methyl-1H-indol-2-yl)ethan-1-ol **8** was used without further purification.

A solution of compound **8** (440 mg, 2.51 mmol), triphenylphosphine (730 mg, 2.76 mmol), 4-(methylthio)phenol **9a** (400 mg, 2.76 mmol), and diisopropyl azodicarboxylate (DIAD) (0.56 mL, 2.76 mmol) in tetrahydrofuran (15 mL) was stirred at rt for 24 h. The reaction was partitioned sat $NaHCO_3$ (40 mL) and EtOAc (40 mL). The two layers were separated and the organic layer was washed with sat $NaHCO_3$ (30 mL), and brine (30 mL) and dried over Na_2SO_4. The solvent was evaporated off and the crude was purified on silica gel chromatography, eluting with EtOAc/Hexanes 1:9 to give **10** as a white solid. Yield = 315 mg (42 %). ^1H NMR (700 MHz, $CDCl_3$) δ 7.61 (d, J = 7.8 Hz, 1H), 7.33 (d, J = 8.3 Hz, 1H), 7.23 (m, 3H), 7.12 (t, J = 7.5 Hz, 1H), 6.94 (d, J = 8.9 Hz, 2H), 6.56 (s, 1H), 5.61 – 5.58 (m, 1H), 3.79 (s, 3H), 2.45 (s, 3H), 1.82 (d, J = 6.5 Hz, 3H). ^{13}C NMR (176 MHz, $CDCl_3$) δ 155.9, 139.4, 138.1, 129.9, 129.6, 127.2, 121.9, 120.8,119.6, 116.7, 109.1, 100.6, 69.8, 30.4, 20.1, 17.8. HRMS (EI) m/z Calcd. for $C_{18}H_{19}OSN$ $[M + H]^+$: 298.1260, found 298.1256.

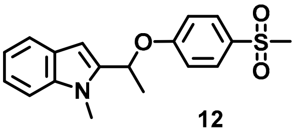

12

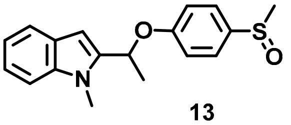

13

1-methyl-2-(1-(4-(methylsulfonyl)phenoxy)ethyl)-1H-indole (**12**) and 1-methyl-2-(1-(4-(methylsulfinyl)phenoxy)ethyl)-1H-indole (**13**): To a solution of **10** (70 mg, 0.24 mmol), diisopropylamine (6.6 µL, 0.05 mmol), in CH_3CN (1 mL) was added drop wisely to a solution of oxone (232 mg, 0.38 mmol) in water (2.0 mL). Stirring continued at rt and a precipitate developed. Ice cold water (15 mL) was added and the suspension was filtered. The residue was washed with cold water (30 mL) and air dried. TLC of the dried solid revealed a quantitative formation of 10 and the formation of two new products (rfs 0.77 and 0.45, CH_2Cl_2/MeOH 14:1). The crude was purified with prep-TLC, eluting with CH_2Cl_2/MeOH 20:1 to furnish **12** (rf = 0.77 compound) and **13** (rf = 0.45 compound) as white solid. Yield: compound **12** = 14 mg (18 %); compound **13** = 16 mg (22 %).

Characterization of **12**: ^{1}H NMR (700 MHz, $CDCl_3$) δ 7.85 (d, *J* = 6.7 Hz, 2H), 7.62 (d, *J* = 7.9 Hz, 1H), 7.34 (d, *J* = 8.3 Hz, 1H), 7.28 – 7.24 (m, 3H), 7.12 (d, *J* = 7.9 Hz, 2H), 5.73 (q, *J* = 6.5 Hz, 1H), 3.79 (s, 3H), 3.02 (s, 3H), 1.88 (d, *J* = 6.9 Hz, 3H). ^{13}C NMR (176 MHz, $CDCl_3$) δ 161.7, 138.2, 138.2, 132.7, 129.6, 127.1, 122.2, 120.9, 119.9, 116.0, 109.2, 101.1, 70.3, 44.8, 30.5, 20.4. Calcd. for $C_{18}H_{19}O_3SN$ [M + H]$^+$: 330.1158, found 330.1144.

Characterization of **13**: ^{1}H NMR (700 MHz, $CDCl_3$) δ 7.61 (d, *J* = 8.0 Hz, 1H), 7.57 (dd, *J* = 8.9, 3.1 Hz, 2H), 7.33 (d, *J* = 8.3 Hz, 1H), 7.25 (t, *J* = 7.6 Hz, 2H), 7.13 (d, *J* = 8.8 Hz, 2H), 6.58 (s, 1H), 5.69 (q, *J* = 6.5 Hz, 1H), 3.80 (s, 3H), 2.71 (s, 3H), 1.86 (dd, *J* = 6.6, 3.3 Hz, 3H). ^{13}C NMR (176 MHz, $CDCl_3$) δ 160.0, 159.9, 138.1, 129.7, 127.1, 125.6, 125.6, 122.1, 120.8, 119.8, 116.6, 109.1, 100.9, 100.9, 70.0, 43.9, 30.4, 20.3. Calcd. for $C_{18}H_{19}O_2SN$ [M + H]$^+$: 314.1209, found 314.1198.

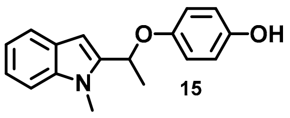

15

4-(1-(1-methyl-1H-indol-2-yl)ethoxy)phenol (**15**): Compound **8** (330 mg, 1.9 mmol), triphenylphosphine (608 mg, 2.30 mmol), 4-((tert-butyldimethylsilyl)oxy)phenol **9b** (516 mg, 2.30 mmol), and diisopropyl azodicarboxylate (DIAD) (0.46 mL, 2.30 mmol) in tetrahydrofuran (10 mL) was stirred at rt for 24 h. The reaction was worked up as described for the synthesis of 10 to furnish the crude compound 11, which was subsequently reacted with CsF (364 mg, 2.4 mmol) in MeOH (20 mL) at rt for 2 h. The reaction was partitioned between water (40 mL) and CH_2Cl_2 (50 mL). The two layers were separated, the aqueous layer was washed with CH_2Cl_2 (40 mL), and the combined organic layer was washed with half-saturated brine (50 mL) and dried over Na_2SO_4. The solvent was evaporated off and the crude was purified on prep-TLC, eluting with EtOAc/Hexanes 1:4 to give **15** as a white solid. ^{1}H NMR (700 MHz, $CDCl_3$) δ 7.63 – 7.57 (d, J = 7.9 Hz, 1H), 7.35 – 7.31 (d, J = 8.3 Hz, 1H), 7.26 – 7.21 (d, J = 8.2 Hz, 1H), 7.15 – 7.10 (t, J = 7.5 Hz, 1H), 6.88 – 6.82 (s, 2H), 6.75 – 6.68 (s, 2H), 6.52 (s, 1H), 5.52 – 5.47 (q, J = 6.6 Hz, 1H), 4.6 (bs, 1H), 3.79 (s, 3H), 1.81 – 1.78 (d, J = 6.6 Hz, 3H). ^{13}C NMR (176 MHz, $CDCl_3$) δ 151.5, 150.0, 139.8, 138.1, 127.2, 121.8, 120.7, 119.5, 117.7, 116.1, 109.1, 100.5, 70.2, 30.4, 20.2. MS [M+H]$^+$: 268.4.

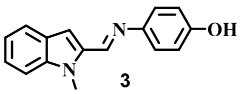

3

AO-03-17/AO-03-17, 1H 400MHz

^{1}H NMR (700 MHz, DMSO) δ 9.52 (s, 1H), 8.71 (s, 1H), 7.64 (d, *J* = 8.4 Hz, 1H), 7.52 (d, *J* = 7.6 Hz, 1H), 7.28 (t, *J* = 7.6 Hz, 1H), 7.24 (d, *J* = 8.5 Hz, 2H), 7.09 (t, *J* = 7.5 Hz, 1H), 7.07 (s, 1H), 6.82 (d, *J* = 8.9 Hz, 2H), 4.17 (s, 3H), 3.35 (s, 1H).

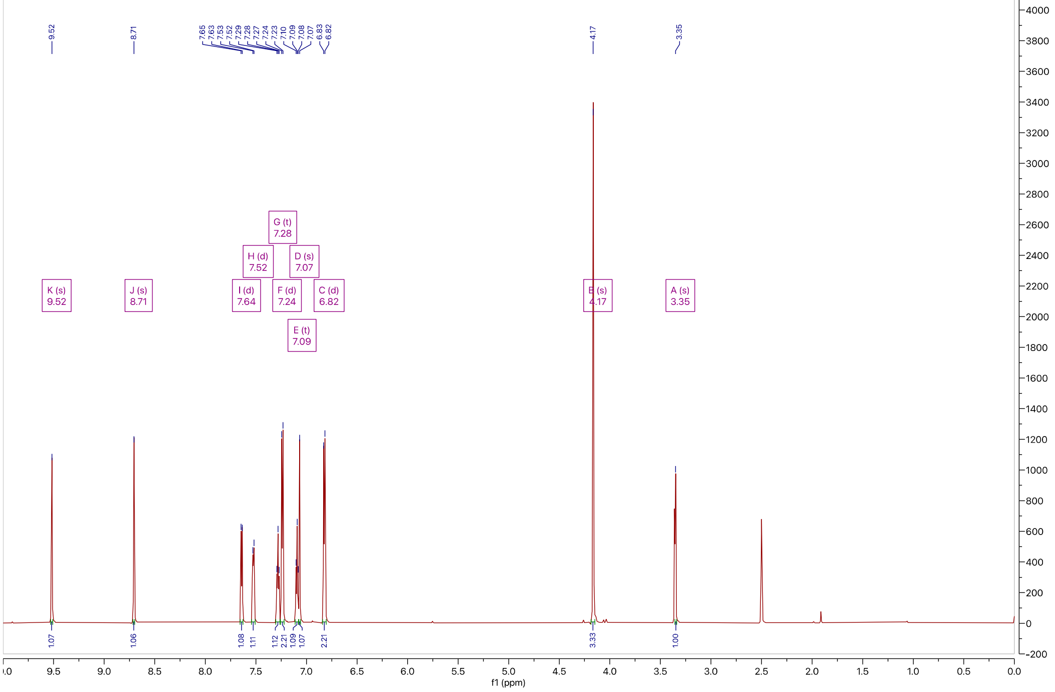

AO-03-17/AO-03-17,13C 700MHz

^{13}C NMR (176 MHz, DMSO) δ 156.50, 149.64, 143.04, 139.88, 136.04, 126.89, 124.16, 122.64, 121.67, 120.26, 115.99, 110.46, 109.70, 31.82.

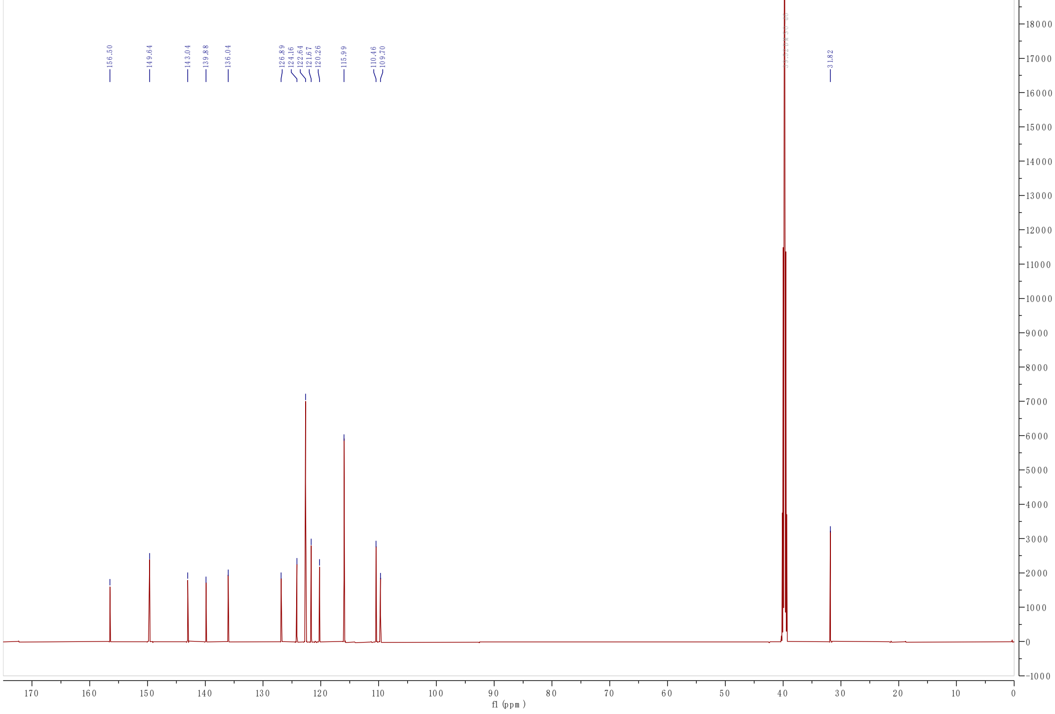

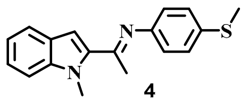

4

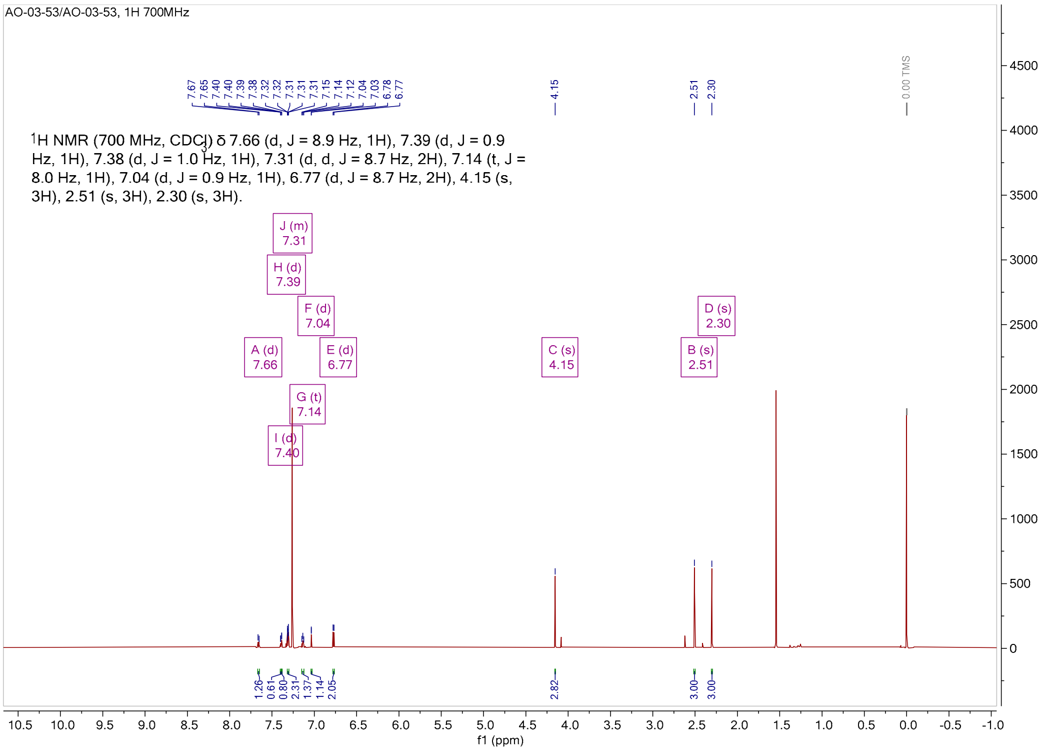

AO-03-53/AO-03-53, 1H 700MHz
1H NMR (700 MHz, CDCl3) δ 7.66 (d, J = 8.9 Hz, 1H), 7.39 (d, J = 0.9 Hz, 1H), 7.38 (d, J = 1.0 Hz, 1H), 7.31 (d, d, J = 8.7 Hz, 2H), 7.14 (t, J = 8.0 Hz, 1H), 7.04 (d, J = 0.9 Hz, 1H), 6.77 (d, J = 8.7 Hz, 2H), 4.15 (s, 3H), 2.51 (s, 3H), 2.30 (s, 3H).
J (m) 7.31
H (d) 7.39
F (d) 7.04
A (d) 7.66
E (d) 6.77
C (s) 4.15
D (s) 2.30
B (s) 2.51
G (t) 7.14
I (d) 7.40
f1 (ppm)

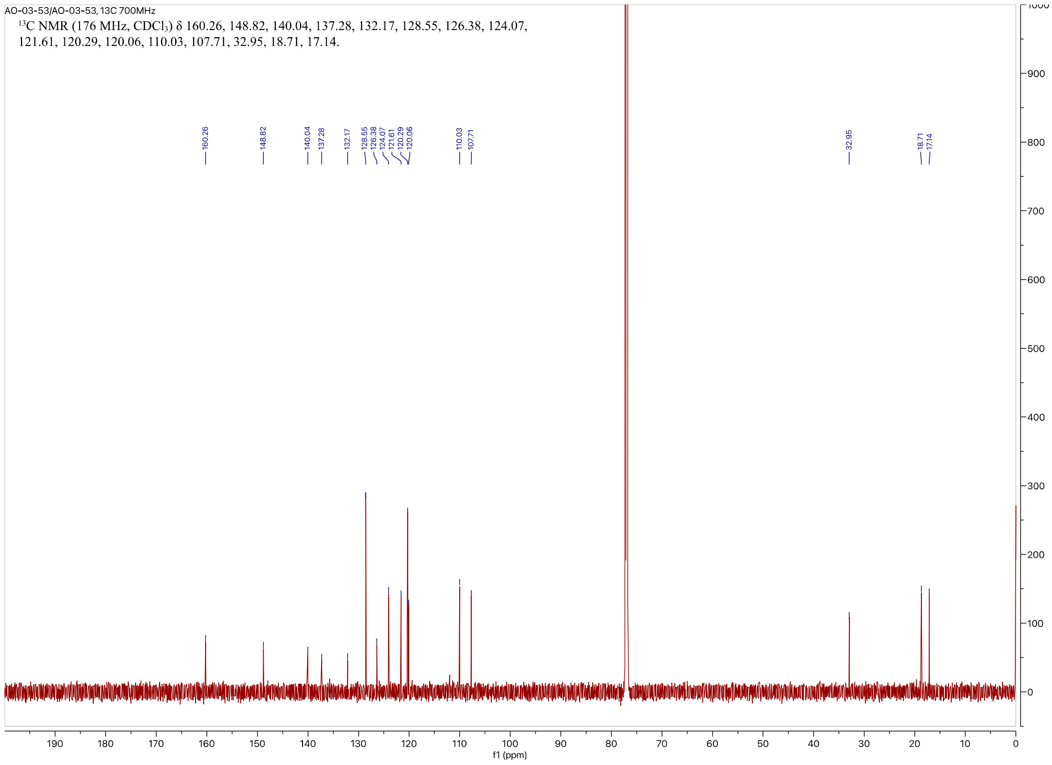

AO-03-53/AO-03-53, 13C 700MHz
^{13}C NMR (176 MHz, $CDCl_3$) δ 160.26, 148.82, 140.04, 137.28, 132.17, 128.55, 126.38, 124.07, 121.61, 120.29, 120.06, 110.03, 107.71, 32.95, 18.71, 17.14.
f1 (ppm)

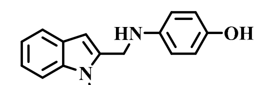
HN
OH
N

5

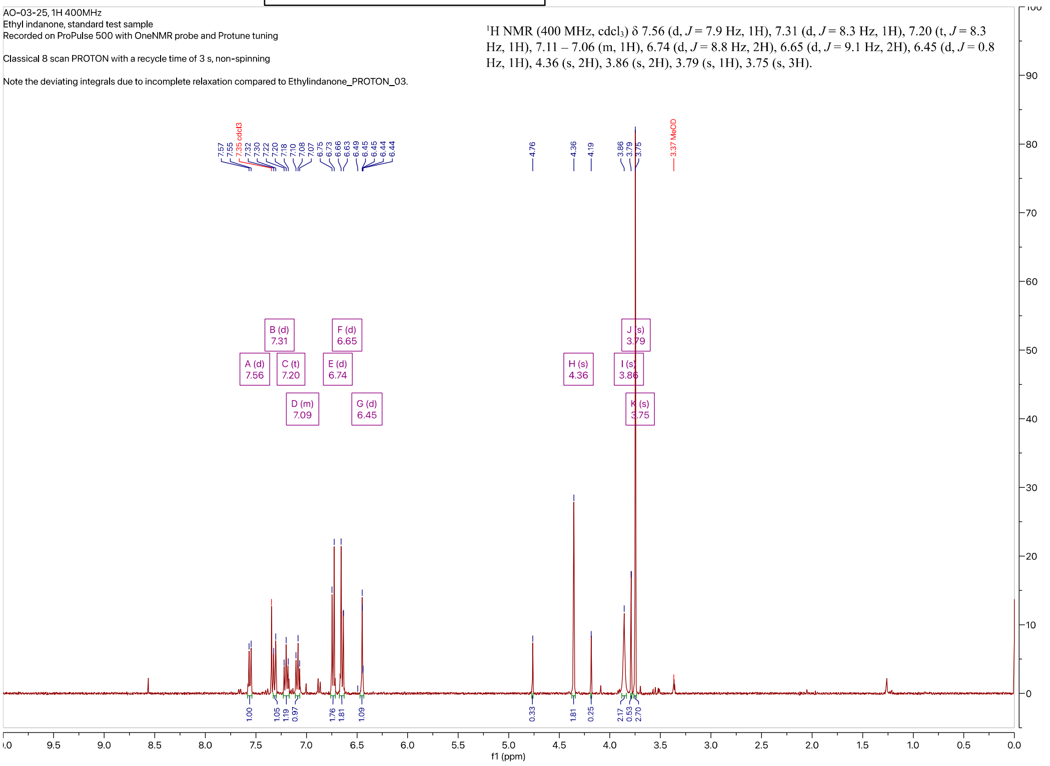
AO-03-25, 1H 400MHz
Ethyl indanone, standard test sample
Recorded on ProPulse 500 with OneNMR probe and Protune tuning
Classical 8 scan PROTON with a recycle time of 3 s, non-spinning
Note the deviating integrals due to incomplete relaxation compared to Ethylindanone_PROTON_03.
¹H NMR (400 MHz, cdcl₃) δ 7.56 (d, J = 7.9 Hz, 1H), 7.31 (d, J = 8.3 Hz, 1H), 7.20 (t, J = 8.3 Hz, 1H), 7.11 – 7.06 (m, 1H), 6.74 (d, J = 8.8 Hz, 2H), 6.65 (d, J = 9.1 Hz, 2H), 6.45 (d, J = 0.8 Hz, 1H), 4.36 (s, 2H), 3.86 (s, 2H), 3.79 (s, 1H), 3.75 (s, 3H).
7.35 cdcl3
3.37 MeOD
f1 (ppm)

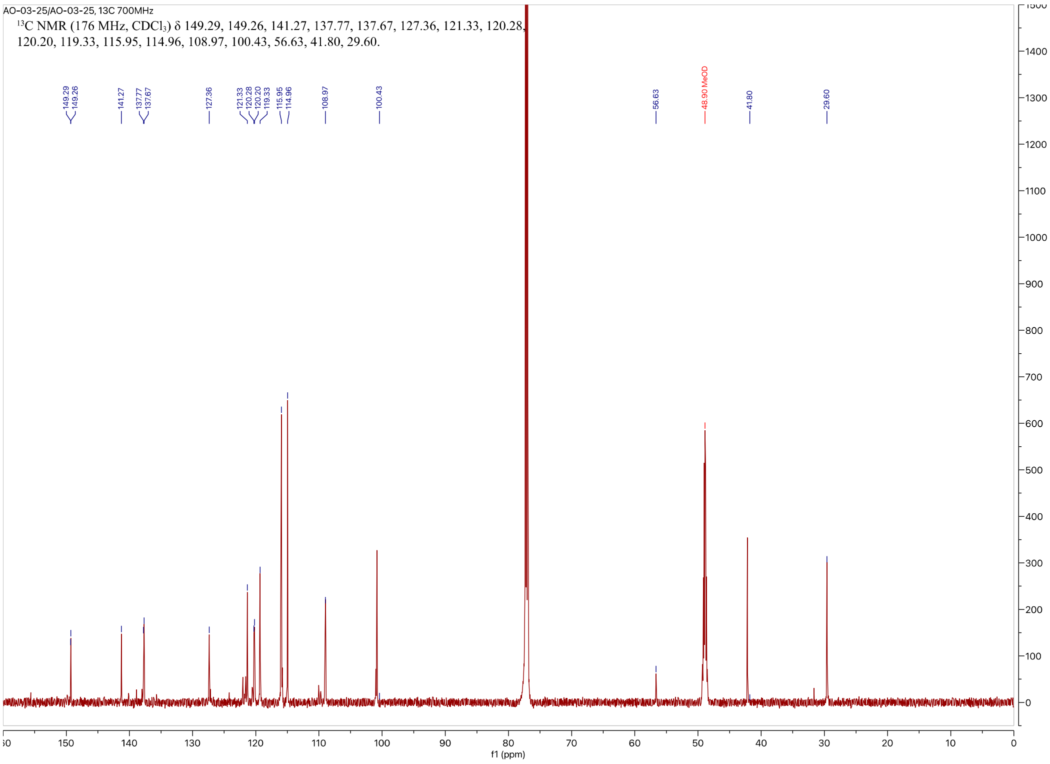
AO-03-25/AO-03-25, 13C 700MHz
¹³C NMR (176 MHz, CDCl₃) δ 149.29, 149.26, 141.27, 137.77, 137.67, 127.36, 121.33, 120.28, 120.20, 119.33, 115.95, 114.96, 108.97, 100.43, 56.63, 41.80, 29.60.
48.90 MeOD
f1 (ppm)

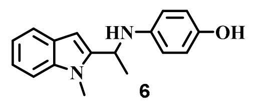
HN
OH
N

6

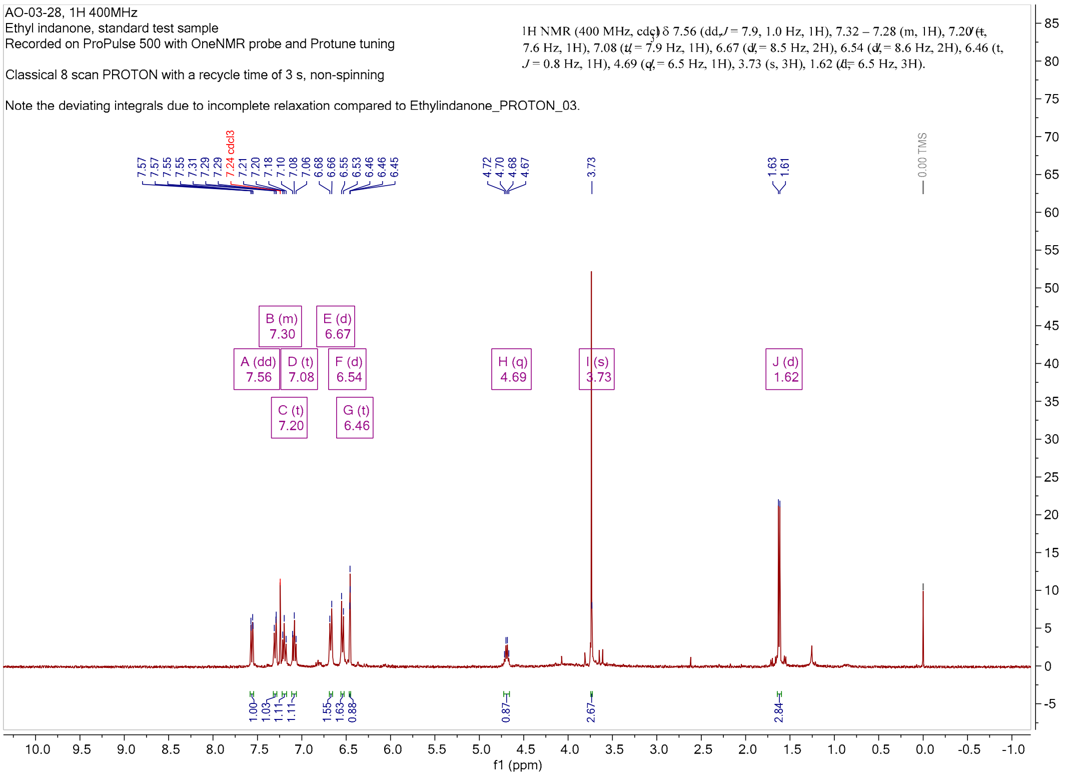
AO-03-28, 1H 400MHz
Ethyl indanone, standard test sample
Recorded on ProPulse 500 with OneNMR probe and Protune tuning
Classical 8 scan PROTON with a recycle time of 3 s, non-spinning
Note the deviating integrals due to incomplete relaxation compared to Ethylindanone_PROTON_03.
1H NMR (400 MHz, cdcl3) δ 7.56 (dd, J = 7.9, 1.0 Hz, 1H), 7.32 – 7.28 (m, 1H), 7.20 (t, J = 7.6 Hz, 1H), 7.08 (t, J = 7.9 Hz, 1H), 6.67 (d, J = 8.5 Hz, 2H), 6.54 (d, J = 8.6 Hz, 2H), 6.46 (t, J = 0.8 Hz, 1H), 4.69 (q, J = 6.5 Hz, 1H), 3.73 (s, 3H), 1.62 (d, J = 6.5 Hz, 3H).
A (dd) 7.56
B (m) 7.30
C (t) 7.20
D (t) 7.08
E (d) 6.67
F (d) 6.54
G (t) 6.46
H (q) 4.69
I (s) 3.73
J (d) 1.62
0.00 TMS
f1 (ppm)

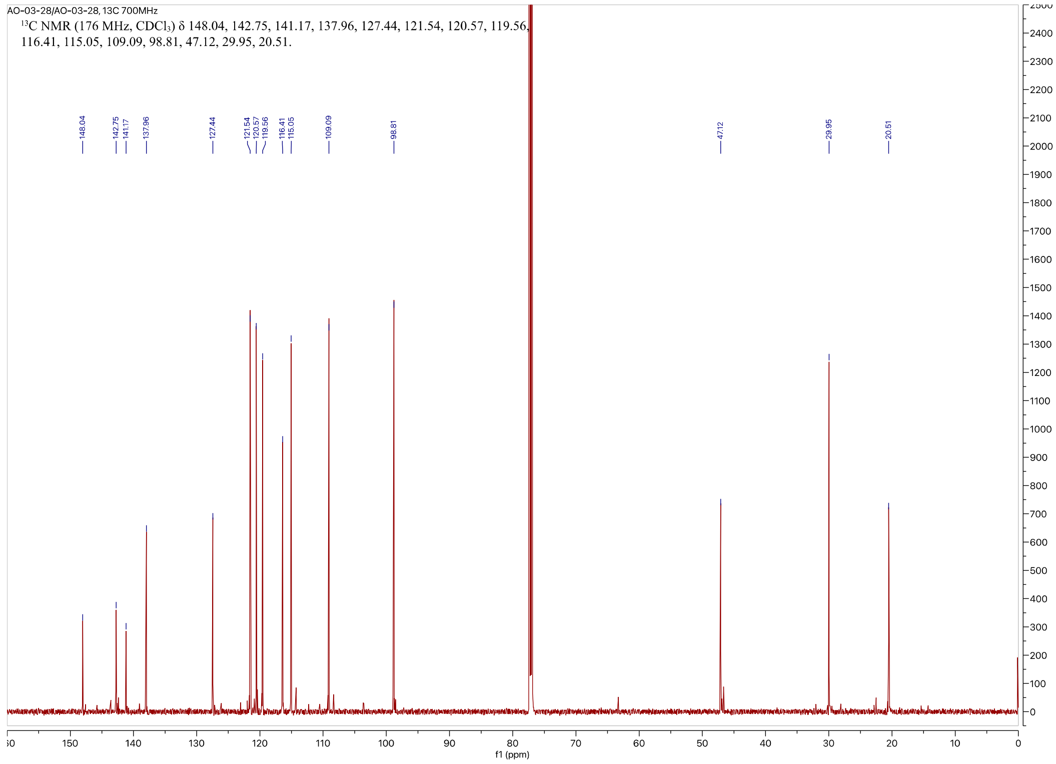
AO-03-28/AO-03-28, 13C 700MHz
13C NMR (176 MHz, CDCl3) δ 148.04, 142.75, 141.17, 137.96, 127.44, 121.54, 120.57, 119.56, 116.41, 115.05, 109.09, 98.81, 47.12, 29.95, 20.51.
f1 (ppm)

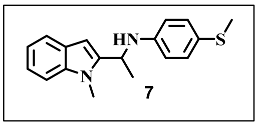
HN
S
N
7

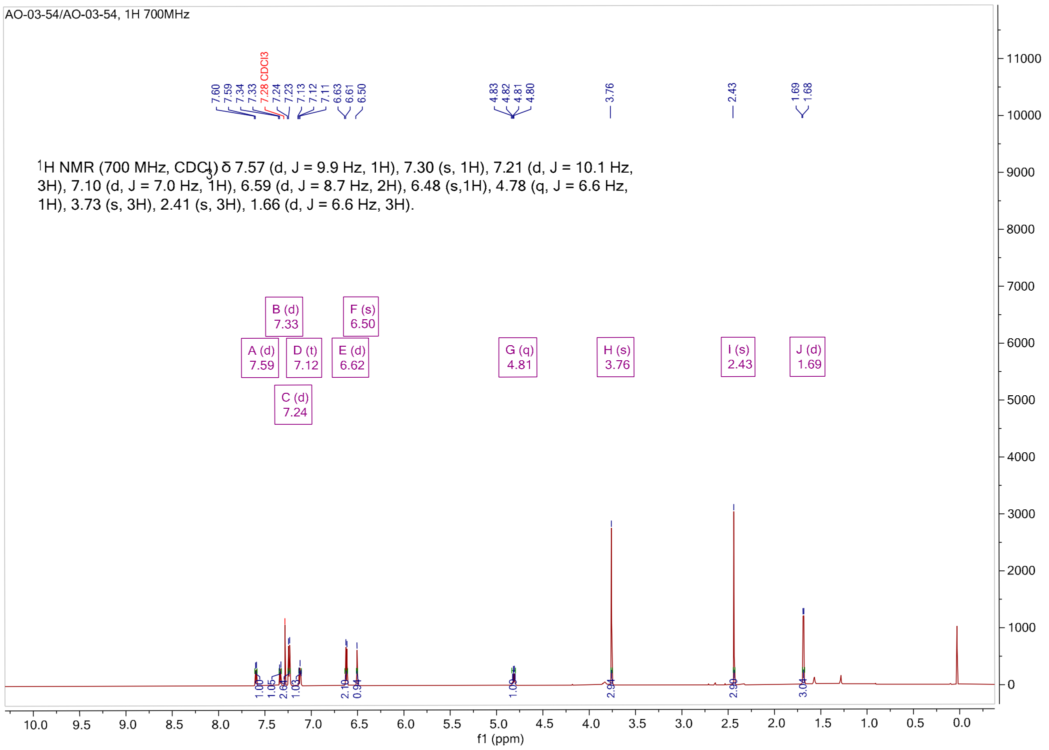
AO-03-54/AO-03-54, 1H 700MHz
1H NMR (700 MHz, CDCl3) δ 7.57 (d, J = 9.9 Hz, 1H), 7.30 (s, 1H), 7.21 (d, J = 10.1 Hz, 3H), 7.10 (d, J = 7.0 Hz, 1H), 6.59 (d, J = 8.7 Hz, 2H), 6.48 (s,1H), 4.78 (q, J = 6.6 Hz, 1H), 3.73 (s, 3H), 2.41 (s, 3H), 1.66 (d, J = 6.6 Hz, 3H).
A (d) 7.59
B (d) 7.33
C (d) 7.24
D (t) 7.12
E (d) 6.62
F (s) 6.50
G (q) 4.81
H (s) 3.76
I (s) 2.43
J (d) 1.69
f1 (ppm)

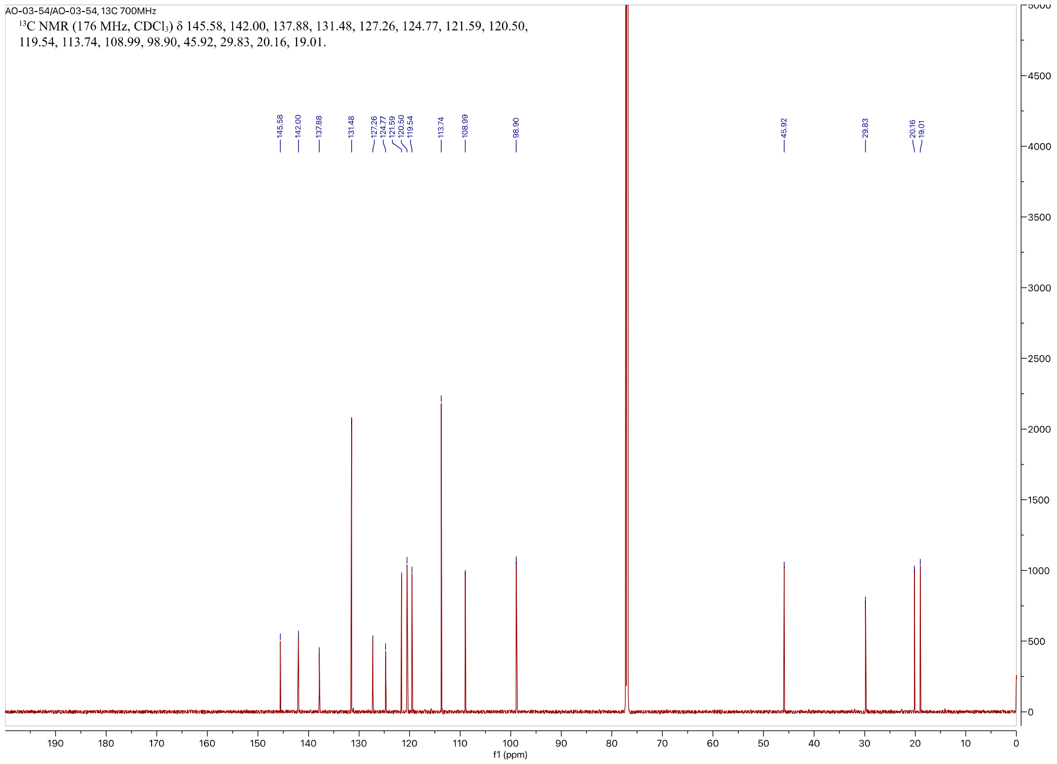
AO-03-54/AO-03-54, 13C 700MHz
13C NMR (176 MHz, CDCl3) δ 145.58, 142.00, 137.88, 131.48, 127.26, 124.77, 121.59, 120.50, 119.54, 113.74, 108.99, 98.90, 45.92, 29.83, 20.16, 19.01.
f1 (ppm)

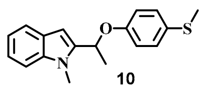

10

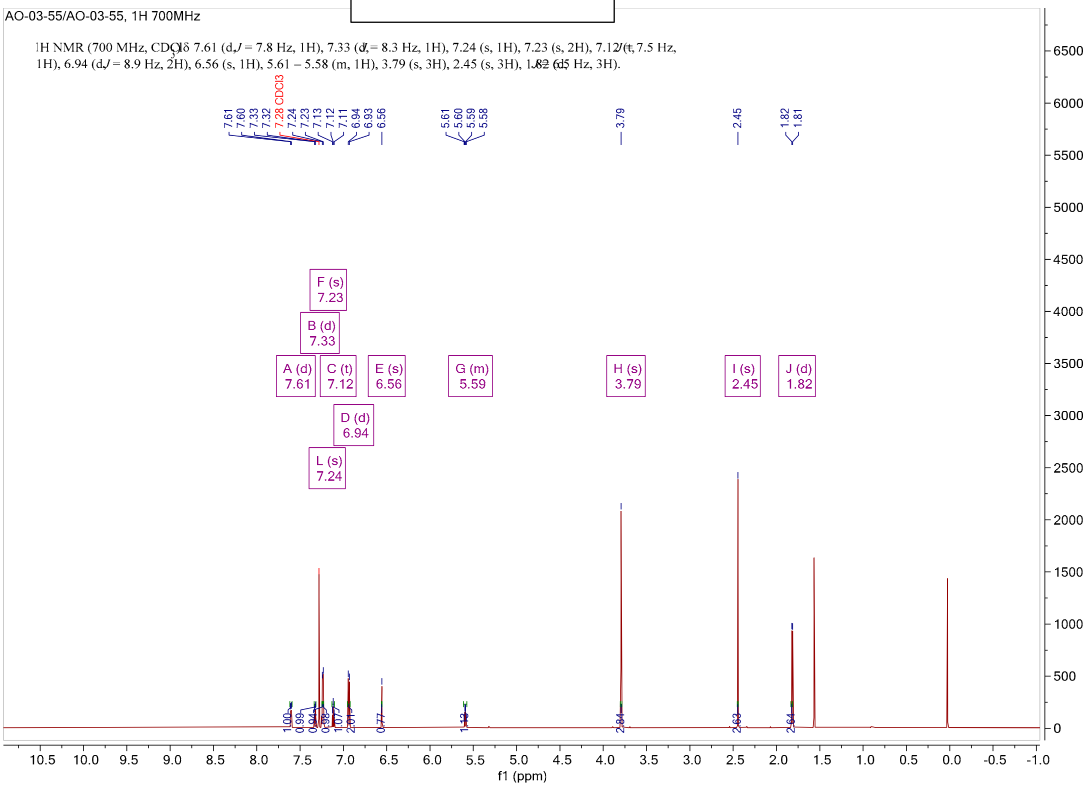
AO-03-55/AO-03-55, 1H 700MHz
1H NMR (700 MHz, CDCl3) δ 7.61 (d, J = 7.8 Hz, 1H), 7.33 (d, J = 8.3 Hz, 1H), 7.24 (s, 1H), 7.23 (s, 2H), 7.12 (t, J = 7.5 Hz, 1H), 6.94 (d, J = 8.9 Hz, 2H), 6.56 (s, 1H), 5.61 – 5.58 (m, 1H), 3.79 (s, 3H), 2.45 (s, 3H), 1.82 (d, J = 6.5 Hz, 3H).
F (s) 7.23
B (d) 7.33
A (d) 7.61
C (t) 7.12
E (s) 6.56
G (m) 5.59
H (s) 3.79
I (s) 2.45
J (d) 1.82
D (d) 6.94
L (s) 7.24
f1 (ppm)

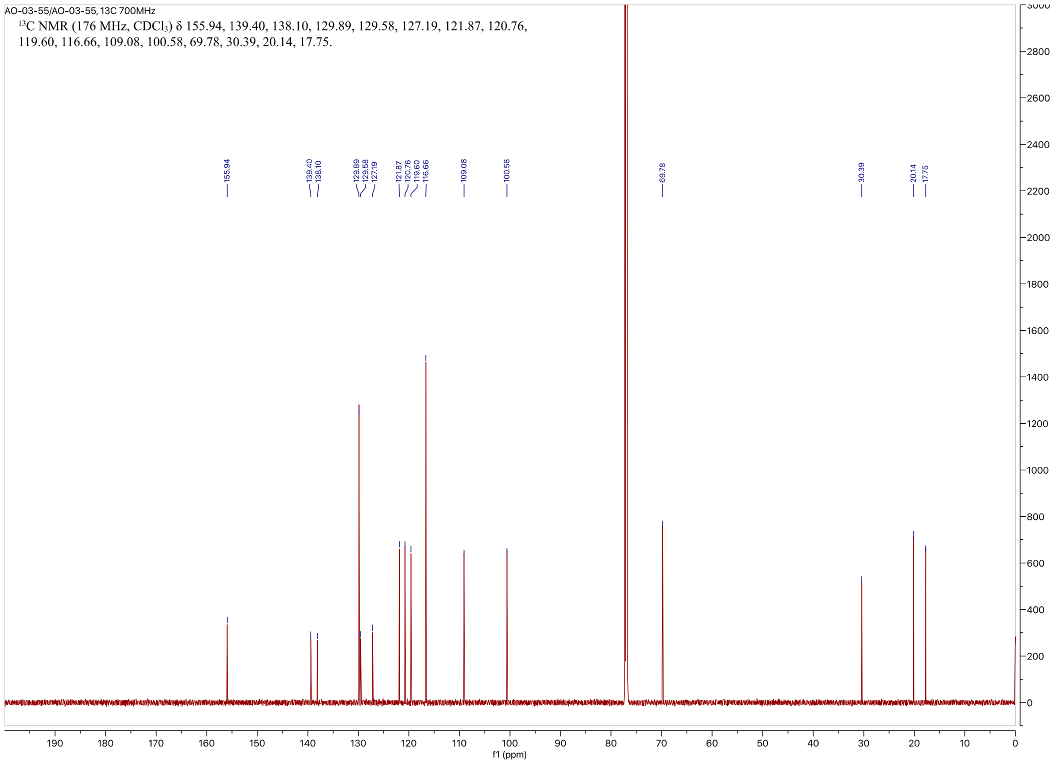
AO-03-55/AO-03-55, 13C 700MHz
13C NMR (176 MHz, CDCl3) δ 155.94, 139.40, 138.10, 129.89, 129.58, 127.19, 121.87, 120.76, 119.60, 116.66, 109.08, 100.58, 69.78, 30.39, 20.14, 17.75.
f1 (ppm)

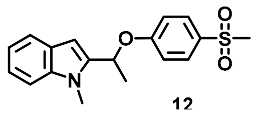

12

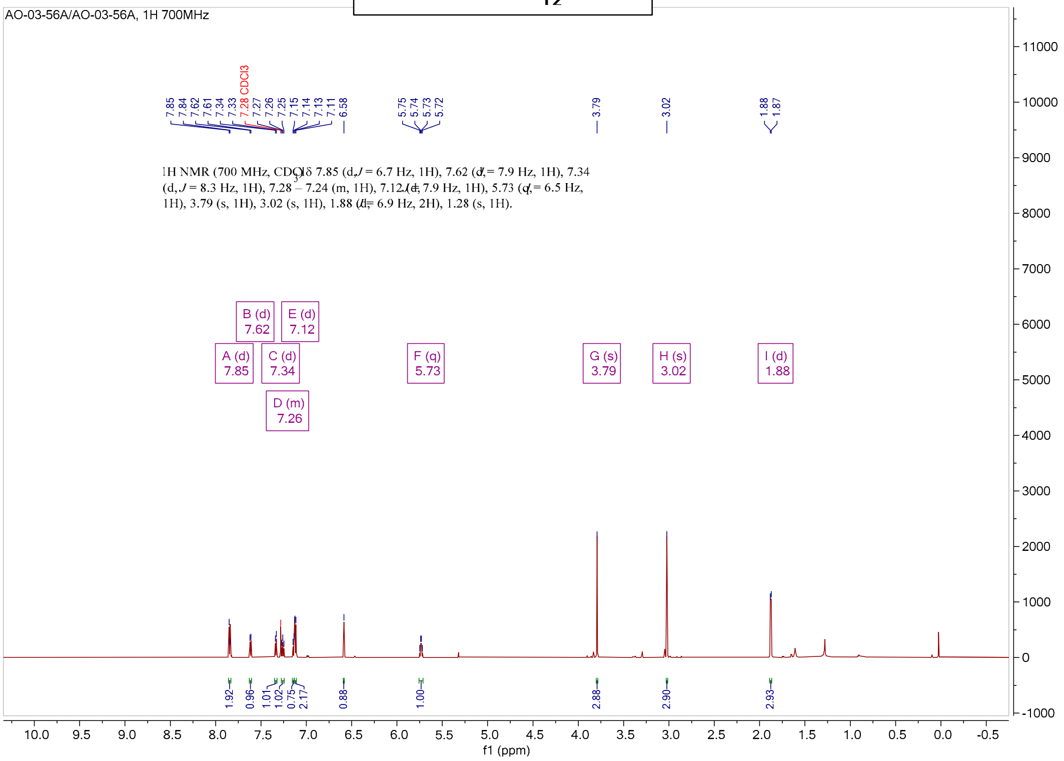
AO-03-56A/AO-03-56A, 1H 700MHz
7.85 7.84 7.62 7.61 7.34 7.33 7.28 CDCl3 7.27 7.26 7.25 7.15 7.14 7.13 7.11 6.58
5.75 5.74 5.73 5.72
3.79
3.02
1.88 1.87
1H NMR (700 MHz, CDCl3) δ 7.85 (d, J = 6.7 Hz, 1H), 7.62 (d, J = 7.9 Hz, 1H), 7.34 (d, J = 8.3 Hz, 1H), 7.28 – 7.24 (m, 1H), 7.12 (d, J = 7.9 Hz, 1H), 5.73 (q, J = 6.5 Hz, 1H), 3.79 (s, 1H), 3.02 (s, 1H), 1.88 (d, J = 6.9 Hz, 2H), 1.28 (s, 1H).
A (d) 7.85
B (d) 7.62
C (d) 7.34
D (m) 7.26
E (d) 7.12
F (q) 5.73
G (s) 3.79
H (s) 3.02
I (d) 1.88
1.92 0.96 1.01 1.02 0.75 2.17 0.88 1.00 2.88 2.90 2.93
f1 (ppm)

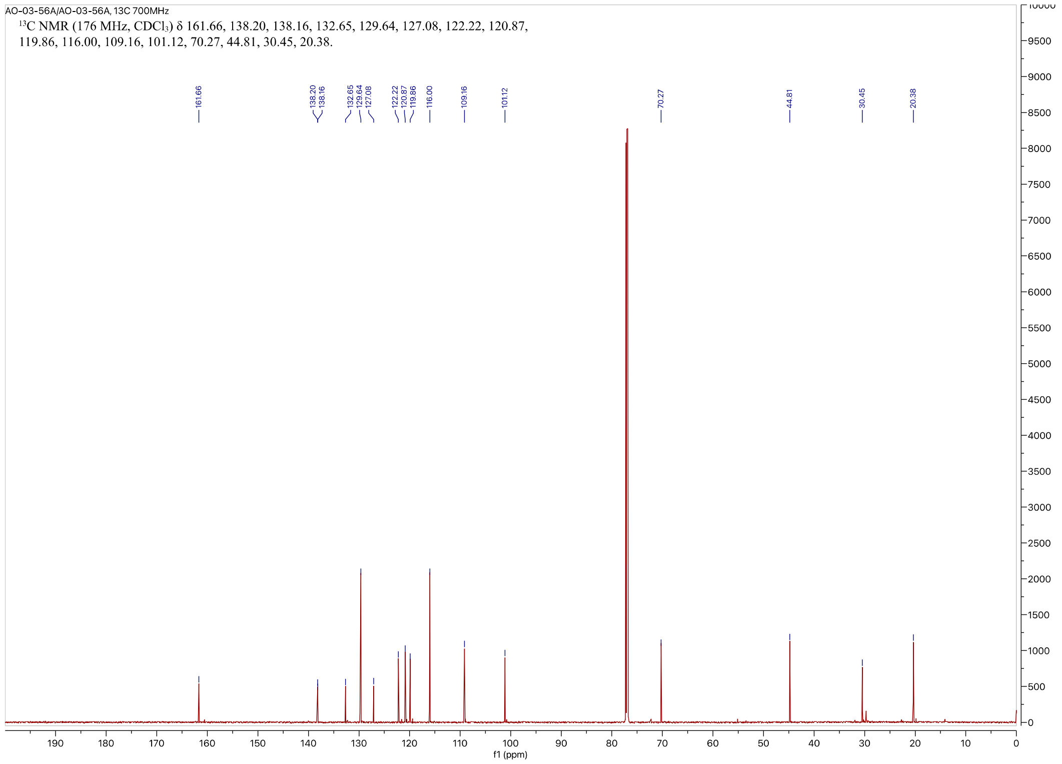
AO-03-56A/AO-03-56A, 13C 700MHz
13C NMR (176 MHz, CDCl3) δ 161.66, 138.20, 138.16, 132.65, 129.64, 127.08, 122.22, 120.87, 119.86, 116.00, 109.16, 101.12, 70.27, 44.81, 30.45, 20.38.
161.66 138.20 138.16 132.65 129.64 127.08 122.22 120.87 119.86 116.00 109.16 101.12 70.27 44.81 30.45 20.38
f1 (ppm)

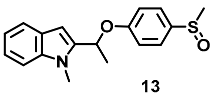

13

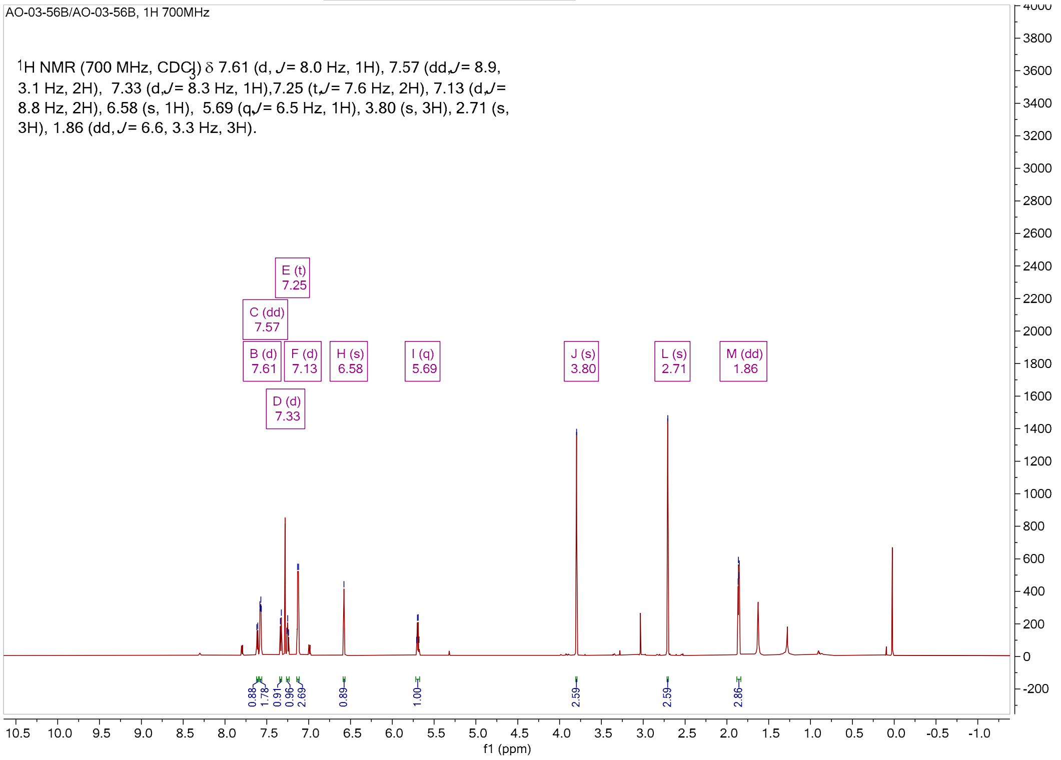
AO-03-56B/AO-03-56B, 1H 700MHz
1H NMR (700 MHz, CDCl3) δ 7.61 (d, J = 8.0 Hz, 1H), 7.57 (dd, J = 8.9, 3.1 Hz, 2H), 7.33 (d, J = 8.3 Hz, 1H), 7.25 (t, J = 7.6 Hz, 2H), 7.13 (d, J = 8.8 Hz, 2H), 6.58 (s, 1H), 5.69 (q, J = 6.5 Hz, 1H), 3.80 (s, 3H), 2.71 (s, 3H), 1.86 (dd, J = 6.6, 3.3 Hz, 3H).
E (t) 7.25
C (dd) 7.57
B (d) 7.61
F (d) 7.13
H (s) 6.58
I (q) 5.69
J (s) 3.80
L (s) 2.71
M (dd) 1.86
D (d) 7.33
0.88
1.78
0.91
0.96
2.69
0.89
1.00
2.59
2.59
2.86
f1 (ppm)

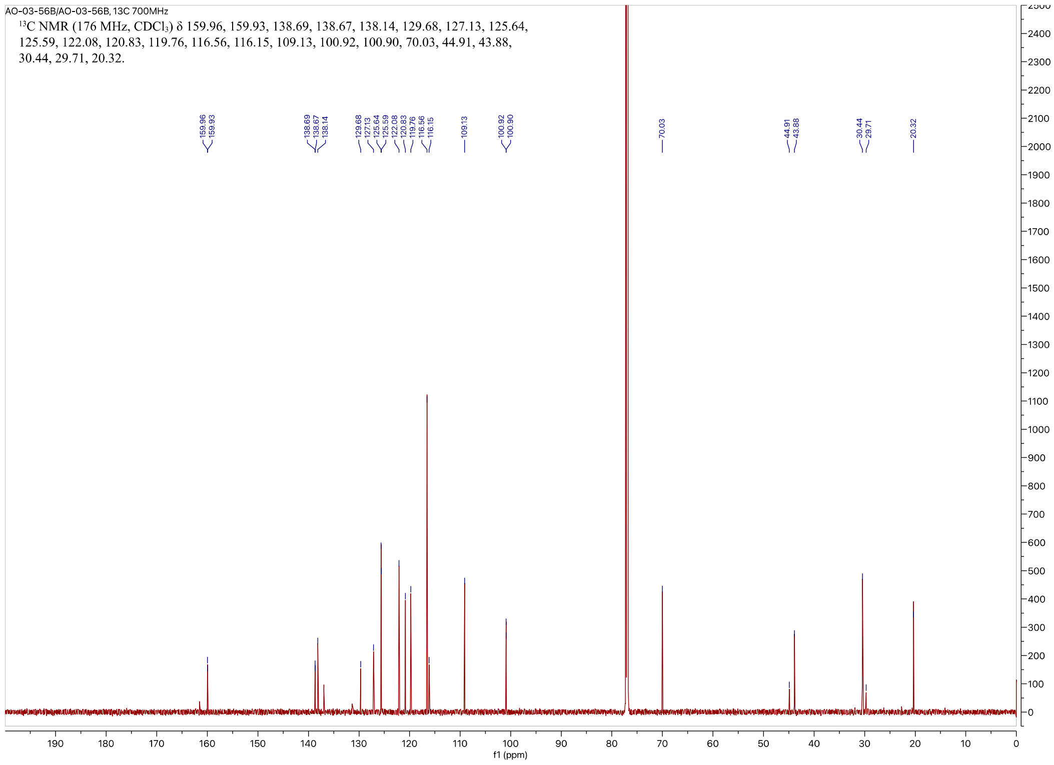
AO-03-56B/AO-03-56B, 13C 700MHz
13C NMR (176 MHz, CDCl3) δ 159.96, 159.93, 138.69, 138.67, 138.14, 129.68, 127.13, 125.64, 125.59, 122.08, 120.83, 119.76, 116.56, 116.15, 109.13, 100.92, 100.90, 70.03, 44.91, 43.88, 30.44, 29.71, 20.32.
f1 (ppm)

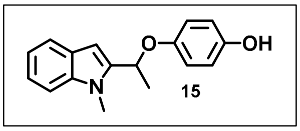
OH
O
N
15

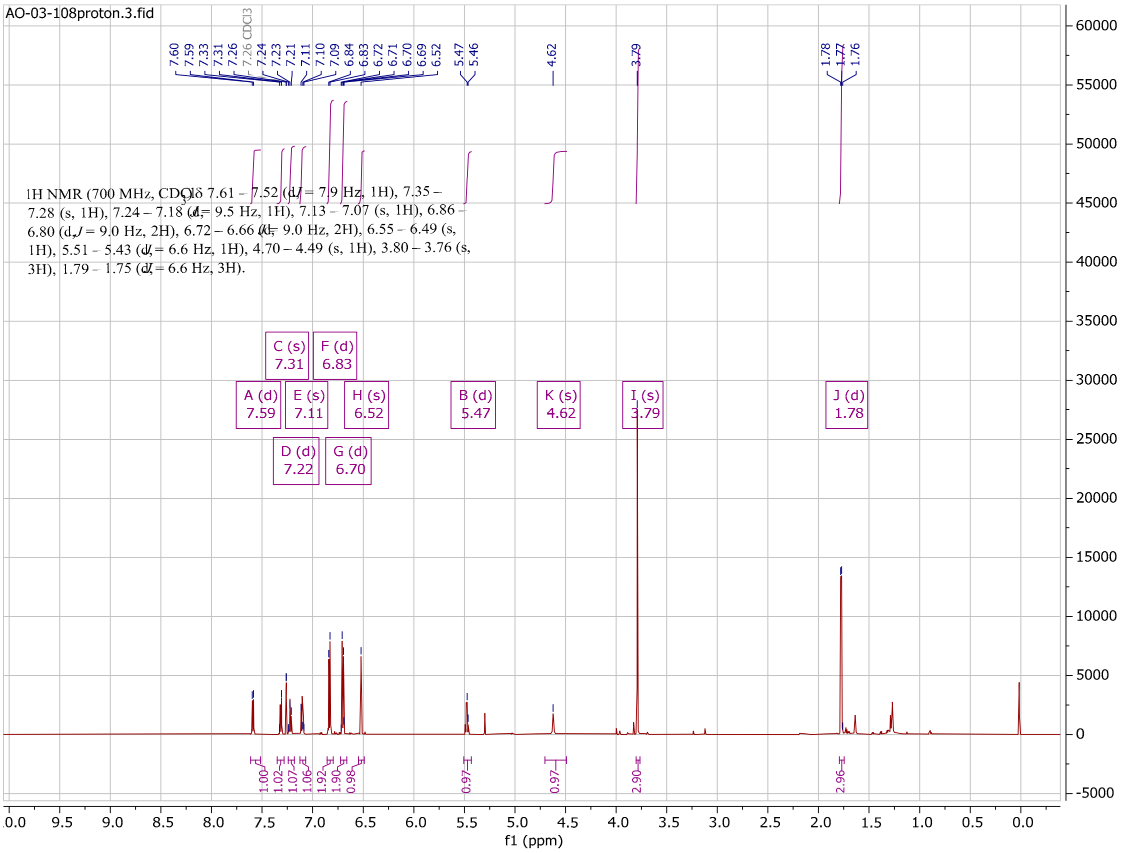
AO-03-108proton.3.fid
1H NMR (700 MHz, CDCl3) δ 7.61 – 7.52 (d, J = 7.9 Hz, 1H), 7.35 – 7.28 (s, 1H), 7.24 – 7.18 (d, J = 9.5 Hz, 1H), 7.13 – 7.07 (s, 1H), 6.86 – 6.80 (d, J = 9.0 Hz, 2H), 6.72 – 6.66 (d, J = 9.0 Hz, 2H), 6.55 – 6.49 (s, 1H), 5.51 – 5.43 (d, J = 6.6 Hz, 1H), 4.70 – 4.49 (s, 1H), 3.80 – 3.76 (s, 3H), 1.79 – 1.75 (d, J = 6.6 Hz, 3H).
7.60 7.59 7.33 7.31 7.26 7.26 CDCl3 7.24 7.23 7.21 7.11 7.10 7.09 6.84 6.83 6.72 6.71 6.70 6.69 6.52 5.47 5.46 4.62 3.79 1.78 1.77 1.76
A (d) 7.59
B (d) 5.47
C (s) 7.31
D (d) 7.22
E (s) 7.11
F (d) 6.83
G (d) 6.70
H (s) 6.52
I (s) 3.79
J (d) 1.78
K (s) 4.62
1.00 1.02 1.07 1.06 1.92 1.90 0.98 0.97 0.97 2.90 2.96
f1 (ppm)

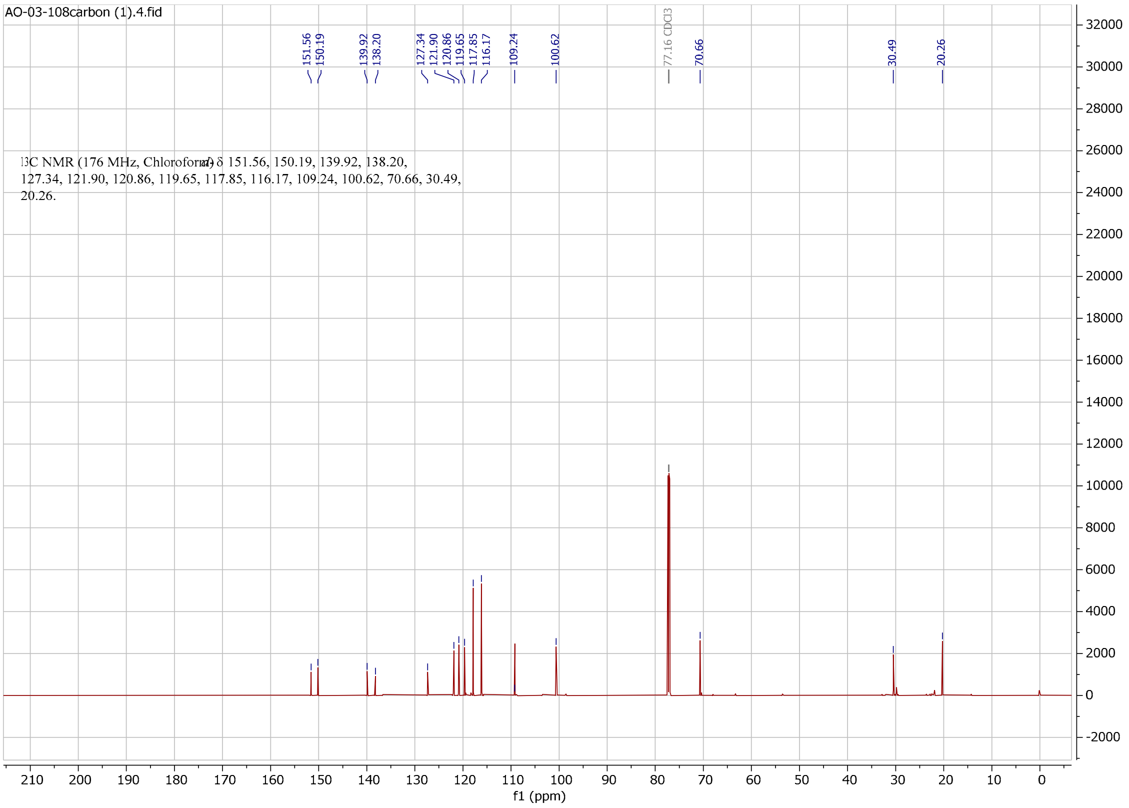
AO-03-108carbon (1).4.fid
13C NMR (176 MHz, Chloroform-d) δ 151.56, 150.19, 139.92, 138.20, 127.34, 121.90, 120.86, 119.65, 117.85, 116.17, 109.24, 100.62, 70.66, 30.49, 20.26.
151.56 150.19 139.92 138.20 127.34 121.90 120.86 119.65 117.85 116.17 109.24 100.62 77.16 CDCl3 70.66 30.49 20.26
f1 (ppm)
